# Epigenetic repurposing of carbohydrate metabolic inhibitors for insulin resistance: targeting DNMT1 and HDAC3 for β-cell restoration

**DOI:** 10.3389/freae.2025.1691949

**Published:** 2025-10-30

**Authors:** Muhammad Javid Iqbal, Luis A. Salazar

**Affiliations:** ^1^ Doctoral Program in Sciences, Specialization in Applied Cellular and Molecular Biology, Universidad de La Frontera, Temuco, Chile; ^2^ Department of Basic Sciences, Center of Molecular Biology and Pharmacogenetics, Faculty of Medicine, Universidad de La Frontera, Temuco, Chile

**Keywords:** insulin resistance, epigenetics, DNMT1, HDAC3, metabolic enzyme inhibitors, cross-target therapeutics, natural products, computational screening

## Abstract

Insulin resistance, a global metabolic crisis affecting a substantial portion of the world’s population, involves complex metabolic-epigenetic crosstalk that current therapies fail to address. DNA methyltransferase 1 (DNMT1) and histone deacetylase 3 (HDAC3) progressively silence insulin signaling genes, creating a self-perpetuating cycle of metabolic dysfunction. We present a hypothetical cross-target repurposing strategy leveraging established α-amylase and α-glucosidase inhibitors as potential epigenetic modulators. Through systematic computational screening of 100 natural metabolic enzyme inhibitors against DNMT1 and HDAC3 crystal structures (PBD ID: 3PTA, 4A69), we identified ten dual-target candidates with binding affinities ranging from −8.1 to −10.2 kcal/mol. Kotalanol emerged as the lead compound, demonstrating strong binding to both HDAC3 (−9.8 kcal/mol) and DNMT1 (−10.2 kcal/mol). Molecular docking revealed that polyphenolic metabolic inhibitors share structural features enabling interaction with epigenetic enzyme active sites, particularly zinc-binding motifs and aromatic pockets. ADMET profiling confirmed favorable pharmacokinetic properties for the top candidates. Clinically validated compounds including berberine, curcumin, and EGCG provide proof-of-concept for dual metabolic-epigenetic activity. This repurposing approach offers significant advantages: utilizing compounds with established safety profiles, addressing multiple pathogenic mechanisms simultaneously, and accelerating therapeutic development. By targeting both immediate glucose control and long-term epigenetic preservation, these dual-action compounds could transform diabetes management from symptomatic treatment to mechanistic intervention, potentially reversing insulin resistance progression rather than merely managing hyperglycemia.

## 1 Introduction

Insulin resistance is a growing global threat to public health, affecting 46.5% of adults worldwide (Fahed et al., 2020). This metabolic dysfunction drives the type 2 diabetes epidemic, projected to affect 783 million people by 2045 (Hossain et al., 2024). Decades of therapeutic development have produced treatments like metformin, thiazolidinediones, and GLP-1 receptor agonists. However, these drugs only provide symptomatic management. They fail to reverse the fundamental molecular dysfunction underlying this metabolic disorder (Gier et al., 2025). Current approaches target downstream metabolic pathways while overlooking the epigenetic mechanisms that silence insulin signaling genes. These mechanisms involve DNA methyltransferase 1 (DNMT1) and histone deacetylase 3 (HDAC3) dysregulation. This oversight creates a critical gap in our therapeutic strategy. Even well-managed patients receiving optimal standard care continue to show insulin resistance progression (Min et al., 2017).

While multiple DNA methyltransferases and histone deacetylases exist, we specifically focus on DNMT1 and HDAC3 based on their unique roles in metabolic regulation. DNMT1, unlike *de novo* methyltransferases DNMT3A/3B, is the maintenance methyltransferase responsible for perpetuating methylation patterns during cell division, making it critical for the progressive nature of insulin resistance. Among the 18 mammalian HDACs, HDAC3 uniquely functions as the catalytic component of nuclear receptor co-repressor complexes (NCoR/SMRT) that specifically regulate metabolic gene expression in liver, muscle, and adipose tissue. Furthermore, tissue-specific deletion studies demonstrate that HDAC3, but not other HDAC isoforms, is essential for maintaining metabolic homeostasis (Sun et al., 2012; Chen et al., 2016).

Current insulin resistance therapeutics operate through isolated molecular mechanisms. They address individual pathway components rather than systemic dysfunction. Metformin primarily activates adenosine monophosphate-activated protein kinase (AMPK). This enhances glucose uptake and reduces hepatic glucose production. Yet this mechanism does not restore silenced insulin receptor expression (Apostolova et al., 2020). Thiazolidinediones function as peroxisome proliferator-activated receptor gamma (PPAR-γ) agonists. They improve adipocyte insulin sensitivity. But they cannot reverse the hypermethylation of insulin signaling genes in muscle and liver tissues (Quinn et al., 2008). Similarly, sodium-glucose cotransporter-2 (SGLT2) inhibitors reduce glucose reabsorption in kidneys. They do not address the epigenetic silencing of glucose transporter 4 (GLUT4) expression in peripheral tissues (Bays, 2013). Most significantly, none of these established therapeutic classes directly target DNMT1-mediated hypermethylation. They also ignore HDAC3-driven deacetylation. These processes progressively silence insulin receptor substrate 1 (IRS-1), phosphatidylinositol 3-kinase (PI3K), and downstream insulin signaling components. This mechanistic limitation explains why current treatments require continuous administration. They often lose efficacy over time as epigenetic silence continues unchecked (Odimegwu et al., 2024).

Cancer research has developed extensive libraries of DNMT1 and HDAC3 inhibitors, with compounds like 5-azacytidine and trichostatin A demonstrating powerful gene reactivation capabilities in oncological applications (Laranjeira et al., 2023). Simultaneously, diabetes research has characterized numerous metabolic enzyme inhibitors, particularly α-amylase and α-glucosidase inhibitors such as acarbose and miglitol, which effectively reduce postprandial glucose excursions (Lam et al., 2023). However, these two research domains have operated in complete isolation, with no systematic investigation of potential dual-target activities. Oncology studies focus exclusively on cell cycle regulation and apoptosis pathways, while diabetes research concentrates solely on carbohydrate metabolism and insulin signaling (Olatunde et al., 2021). This disciplinary separation has prevented researchers from recognizing that many natural products with established metabolic enzyme inhibition may possess previously uncharacterized epigenetic modulatory activities. The absence of cross-domain collaboration has resulted in missed opportunities to identify compounds that could simultaneously inhibit digestive enzymes and reverse epigenetic gene silencing.

This comprehensive review proposes a novel cross-target repurposing framework that could transform how we approach insulin resistance treatment. By systematically examining the interconnected nature of metabolic dysfunction and epigenetic dysregulation, specifically DNMT1-mediated hypermethylation and HDAC3-catalyzed deacetylation, we explore the theoretical potential of established metabolic enzyme inhibitors to serve as epigenetic modulators. Through computational screening methodologies applied to natural α-amylase and α-glucosidase inhibitors, we identify structural features that may enable dual-target activity against both metabolic and epigenetic enzymes. This review synthesizes current knowledge across traditionally isolated research domains, establishing criteria for prioritizing compounds based on molecular docking predictions, pharmacokinetic properties, and existing safety profiles. We outline a systematic framework for future experimental validation and discuss how this repurposing strategy could accelerate therapeutic development by leveraging compounds with established clinical histories. By bridging the gap between metabolic and epigenetic therapeutics, this approach offers a potential pathway toward addressing the root causes of insulin resistance rather than merely managing its symptoms.

## 2 Epigenetic basis of insulin resistance

### 2.1 DNMT1 in insulin resistance

DNA methyltransferase 1 (DNMT1) plays a central role in insulin resistance development through systematic hypermethylation of key insulin signaling genes (Ahmed et al., 2020; Kaimala et al., 2023). The enzyme catalyzes the addition of methyl groups to cytosine residues in CpG dinucleotides within gene promoter regions. This modification creates transcriptionally silent chromatin states that progressively impair cellular insulin sensitivity across multiple metabolic tissues (Kaimala et al., 2023).

DNMT1 directly targets the insulin receptor gene promoter. Research shows that insulin-resistant patients have much higher methylation levels compared to healthy people (Ahmed et al., 2020). Studies find 2-3-fold increases in methylation at specific sites near the gene start region. This methylation strongly reduces insulin receptor production in muscle tissue. When researchers measured both methylation and receptor levels, they found a clear negative relationship. Higher methylation meant fewer insulin receptors. The effect appears fastest in skeletal muscle, then spreads to liver and fat tissue. Laboratory studies show DNMT1 moves to the insulin receptor gene within hours of high glucose exposure (Bansal and Pinney, 2017).

IRS-1 serves as a critical bridge in insulin signaling pathwaysDNMT1 methylates the IRS-1 gene promoter during insulin resistance development. This methylation cuts IRS-1 production by more than half of muscle cells exposed to high glucose. Since IRS-1 connects insulin receptors to downstream signaling, its loss breaks the insulin response chain (Ho et al., 2008). Animal studies reveal that IRS-1 methylation happens before insulin resistance becomes obvious. The methylation attracts other proteins that keep the gene permanently shut off (Kovacs et al., 2003).

GLUT4 transporters move glucose into muscle and fat cells. DNMT1 methylates GLUT4 gene regions in insulin-resistant tissue. This methylation reduces GLUT4 production significantly (van Gerwen et al., 2023). Patients with insulin resistance show 50–70% less GLUT4 protein in muscle biopsies. Since GLUT4 controls glucose entry into cells, its reduction directly impairs glucose uptake. Detailed gene analysis reveals specific methylation sites that correlate with glucose uptake problems during insulin tolerance tests (Herman et al., 2022).

DNMT1 affects different tissues in coordinated patterns. Figure 1 illustrates this comprehensive epigenetic control system across major metabolic organs.

**FIGURE 1:**
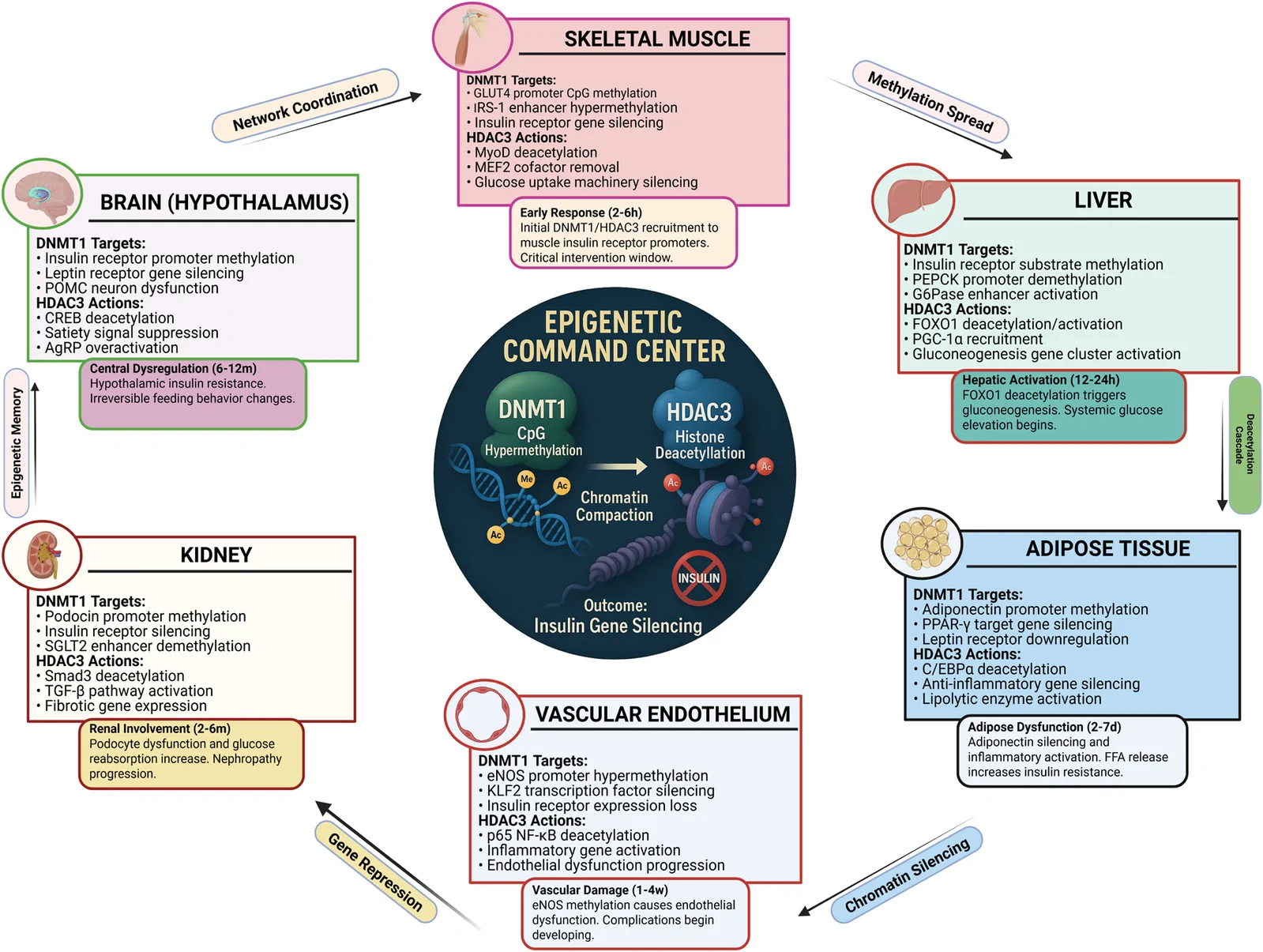
Epigenetic Command Center in Insulin Resistance. DNMT1 and HDAC3 coordinate to silence insulin signaling genes across multiple tissues. The central command center shows how these epigenetic enzymes target specific genes in each tissue type. Skeletal muscle shows GLUT4 and IRS-1 silencing, liver demonstrates gluconeogenesis activation, adipose tissue exhibits inflammatory gene activation, and vascular endothelium shows insulin receptor suppression. Timing annotations indicate early response (2–6 h) in muscle and late effects (12–24 h) in other tissues. This multi-tissue coordination creates systemic insulin resistance through synchronized epigenetic dysfunction (Okuma and Tsuchiya, 2024). Created with BioRender.com, accessed on 12 June 2025.

Skeletal muscle shows the earliest DNMT1 activation. High glucose triggers muscle DNMT1 within 2–4 h. Liver tissue responds differently, with DNMT1 targeting genes that make glucose. Fat tissue develops DNMT1 activity that reduces helpful hormone production. The brain hypothalamus shows DNMT1 effects on appetite control genes. This coordinated response explains why insulin resistance affects the whole body (Ashapkin et al., 2017).

### 2.2 HDAC3 in metabolic dysfunction

Histone deacetylase 3 (HDAC3) emerges as a critical epigenetic regulator that orchestrates metabolic dysfunction through targeted deacetylation of key transcription factors and regulatory proteins. HDAC3 removes acetyl groups from histone tails and non-histone proteins, creating repressive chromatin environments that silence insulin-sensitizing genes while activating gluconeogenic pathways (Zhang and Cao, 2022).

HDAC3 directly targets FOXO1, a protein that controls liver glucose production. When FOXO1 has acetyl groups attached, it stays inactive in the cell cytoplasm. HDAC3 removes these acetyl groups, which activate FOXO1 (Tikhanovich et al., 2013). Active FOXO1 moves into the nucleus and turns on genes that make glucose. These genes include PEPCK and G6Pase, which are key glucose-making enzymes. Studies show HDAC3 activity doubles in insulin-resistant liver tissue. This increased activity correlates with more FOXO1 activation and higher glucose production. Even when insulin levels are high, the liver keeps making glucose because HDAC3 keeps FOXO1 active (Teaney and Cyr, 2023).

HDAC3 shuts down insulin signaling through multiple pathways. The enzyme removes acetyl groups from histones near insulin signaling genes. This makes these genes harder to activate. HDAC3 targets genes for insulin receptors, GLUT4, and IRS-1 across different tissues. Research shows HDAC3 forms complexes with other repressor proteins (Chen et al., 2016). These complexes get recruited to insulin gene promoters during high glucose conditions. HDAC3 activity normally varies throughout the day. It increases during fasting when glucose production should be higher. However, insulin resistance, HDAC3 stays active all the time (Sun et al., 2012).

HDAC3 particularly damages pancreatic cells that make insulin. Figure 2 shows the detailed molecular mechanisms in β-cells.

**FIGURE 2:**
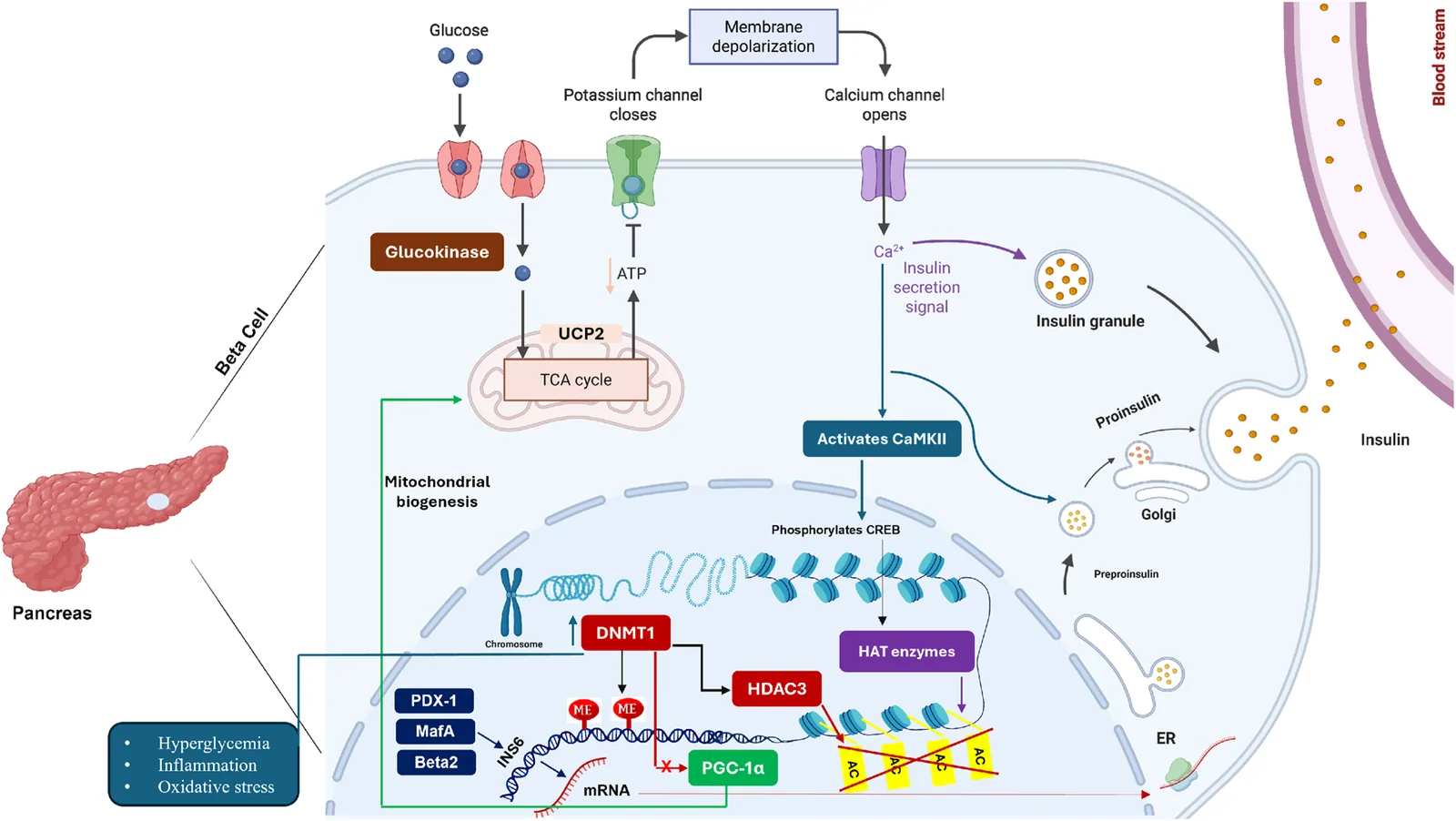
HDAC3-Mediated β-Cell Dysfunction in Insulin Resistance. Pancreatic β-cells show complex HDAC3-driven dysfunction during insulin resistance. Glucose entry triggers calcium signaling normally leading to insulin secretion. However, HDAC3 and DNMT1 interfere with this process by silencing key transcription factors (PDX-1, MafA, Beta2) that control insulin gene expression. HDAC3 deacetylates these transcription factors, reducing their DNA binding and activity. DNMT1 methylates insulin gene promoters, creating permanent silencing. The mitochondrial stress from hyperglycemia further activates these epigenetic enzymes, creating a cycle where β-cells progressively lose insulin production capacity. This explains why insulin deficiency worsens over time in diabetes (Copenhaver and Hoffman, 2017). Created with BioRender.com, accessed on 13 June 2025.

HDAC3 targets essential β-cell proteins including PDX-1, MafA, and NeuroD1. These proteins normally turn on insulin genes. HDAC3 removes acetyl groups from these proteins, making them less effective. Studies in human pancreatic cells show HDAC3 activity increases when glucose levels exceed 10–11 mM. This high activity correlates with reduced insulin content in the cells. HDAC3 also affects β-cell survival by modifying p53 protein. This leads to cell death under high glucose stress. Blocking HDAC3 in diabetic animals restores β-cell function and insulin production (Zhu et al., 2017).

### 2.3 The vicious cycle of epigenetic insulin resistance

DNMT1 and HDAC3 work together to create worsening insulin resistance. This partnership forms a cycle where each round of high glucose makes the next round worse. Understanding this cycle reveals why insulin resistance gets progressively worse over time (Rosen, 2016).

High blood glucose rapidly activates both DNMT1 and HDAC3. This happens within 2–6 h of glucose elevation. Several signals trigger this activation. Advanced glycation products form when glucose attaches to proteins. These products activate inflammatory pathways that turn on DNMT1 and HDAC3 (Negre-Salvayre et al., 2009). Oxidative stress from high glucose also stimulates these enzymes. Research using continuous glucose monitoring shows that glucose spikes matter more than average glucose levels. This suggests that meals causing rapid glucose rises may be particularly harmful (Maude et al., 2021).

Once active, DNMT1 and HDAC3 silence more insulin-related genes. DNMT1 creates permanent methylation marks that stay even after glucose normalizes. HDAC3 provides immediate gene silencing that can reverse quickly but become more stable with repeated activation (Maude et al., 2021). Together, these enzymes silence hundreds of genes involved in glucose metabolism. Studies mapping gene methylation across the genome finds over 300 silenced genes in insulin-resistant patients. These genes control insulin signaling, glucose transport, and cellular energy production (Bansal and Pinney, 2017).

The epigenetic changes create metabolic problems that further activate DNMT1 and HDAC3. Reduced insulin sensitivity leads to higher insulin production from the pancreas. Paradoxically, high insulin activates stress pathways that enhance DNMT1 and HDAC3 activity (Kaimala et al., 2023). Poor glucose uptake keeps blood glucose high, continuing to drive epigenetic enzyme activation. β-cell damage reduces insulin production, leading to even higher glucose levels (Dludla et al., 2023). This explains why insulin resistance typically worsens over the years even with treatment.

The epigenetic nature of this cycle creates opportunities for intervention. Unlike genetic defects, epigenetic changes can potentially be reversed. Several strategies could break this cycle. DNMT1 inhibitors could restore silenced insulin genes (Heerboth et al., 2014). The key insight from our analysis is that compounds targeting both metabolic enzymes and epigenetic regulators could provide superior benefits. Natural products with dual activities offer promise because they have established safety profiles (Atanasov et al., 2021). Early intervention during prediabetes could prevent the establishment of permanent epigenetic dysfunction. This therapeutic rationale underlies our cross-target repurposing approach detailed in subsequent sections.

Accordingly, we centered this review on DNMT1 and HDAC3 because convergent evidence places these enzymes at the core of insulin-resistance epigenetics DNMT1 establishing persistent promoter hypermethylation of insulin-signaling genes and HDAC3 enforcing rapid, recurrent deacetylation-driven repression together sustaining a multi-tissue vicious cycle that is mechanistically actionable.

## 3 Natural product inhibitors of epigenetic regulators

The identification of DNMT1 and HDAC3 as key mediators of epigenetic dysfunction in diabetes, creates specific therapeutic opportunities for intervention. While synthetic inhibitors of these enzymes have been developed primarily for cancer treatment, natural products offer unique advantages including better safety profiles, multiple target engagement, and established traditional use in metabolic disorders. Some natural compounds that can specifically reverse DNMT1-mediated hypermethylation and HDAC3-mediated gene silencing in diabetic pathogenesis are explained.

### 3.1 DNMT1 inhibitors: reversing pathological gene methylation

#### 3.1.1 Berberine: the most clinically advanced DNMT inhibitor

Berberine, an quinoline alkaloid extracted from *Berberis aristata* and other Berberis species, represents the most clinically validated natural DNMT inhibitor for diabetes treatment. This compound targets both DNMT1 and DNMT3B, leading to demethylation and reactivation of silenced metabolic genes. Berberine has advanced to Phase III clinical trials for diabetes management, demonstrating significant improvements in glucose homeostasis (Shrivastava et al., 2023; Camacho et al., 2025).

The mechanism of berberine involves direct binding to DNMT active sites, preventing methylation of insulin and other metabolic genes. Clinical studies have confirmed berberine’s efficacy in reducing HbA1c levels and improving insulin sensitivity, with effects comparable to conventional antidiabetic medications. The compound’s dual action on both DNA methylation and direct metabolic pathways exemplifies the cross-target therapeutic approach central to this review (Belwal et al., 2020).

#### 3.1.2 EGCG: green tea polyphenol with DNMT1 activity

Epigallocatechin gallate (EGCG) from *Camellia sinensis* represents another clinically relevant DNMT1 inhibitor that has progressed to Phase I clinical trials for metabolic syndrome. EGCG achieves DNA demethylation through direct interaction with DNMT1’s catalytic domain, leading to reactivation of genes involved in glucose metabolism and insulin signalling (Belwal et al., 2020; Zwergel et al., 2015).

The therapeutic potential of EGCG extends beyond simple DNMT inhibition to include antioxidant effects and direct metabolic benefits. Studies have demonstrated that EGCG treatment reverses pathological methylation patterns at metabolic gene promoters while simultaneously improving glucose tolerance and reducing oxidative stress in diabetic models (Agarwal et al., 2023).

#### 3.1.3 Additional DNMT1 modulators

Sulforaphane from *Brassica oleracea* (broccoli and other cruciferous vegetables) has reached Phase I clinical trials for diabetes treatment. This compound inhibits both HDAC and DNMT activities while activating the Nrf2 antioxidant pathway, creating multiple beneficial effects for metabolic health. Clinical studies have confirmed sulforaphane’s glucose-lowering effects and its potential for preventing diabetic complications (Kong et al., 2021; Andrés et al., 2025; Myzak et al., 2006).

Emodin, derived from *Rheum palmatum*, demonstrates specific DNMT inhibitory activity in preclinical diabetes models. This anthraquinone compound enhances glucose uptake through demethylation of glucose transporter genes while providing additional metabolic benefits through direct enzyme interactions (Song et al., 2013; Kaleem et al., 2022).

### 3.2 HDAC3 inhibitors: restoring chromatin accessibility

#### 3.2.1 Curcumin: premier natural HDAC inhibitor

Curcumin from *Curcuma longa* stands as the most extensively studied natural HDAC inhibitor for diabetes applications, having advanced to Phase II clinical trials. This polyphenolic compound demonstrates selective inhibition of HDAC1 and HDAC3 while modulating histone acetyltransferase (HAT) activity, creating a balanced approach to chromatin regulation. Clinical studies have confirmed curcumin’s pancreatic β-cell protective effects and its ability to improve glucose tolerance (Liu et al., 2005; Koyu et al., 2024). The mechanism of curcumin involves direct binding to HDAC active sites, particularly HDAC3, leading to increased acetylation of histones at metabolic gene promoters. This results in reactivation of insulin, PDX-1, and other genes silenced during diabetic progression. Studies have demonstrated that curcumin treatment restores glucose-stimulated insulin secretion in diabetic β-cells while providing protective effects against oxidative stress and inflammation (Giommarelli et al., 2010; Dhillon et al., 2008).

#### 3.2.2 Resveratrol: dual SIRT1/HDAC modulator

Resveratrol from *Vitis vinifera* (grape) presents a unique case of dual epigenetic modulation, simultaneously activating SIRT1 while inhibiting other HDAC family members. This compound has reached Phase II clinical trials for diabetes and obesity treatment, demonstrating significant effects on mitochondrial biogenesis and metabolic improvement (Taylor et al., 2008; Karaman Mayack et al., 2020). The dual activity of resveratrol creates complementary effects on cellular metabolism. SIRT1 activation promotes mitochondrial function and fatty acid oxidation, while HDAC inhibition restores expression of metabolic genes silenced during diabetes progression. Clinical studies have confirmed resveratrol’s efficacy in improving glucose tolerance and insulin sensitivity (Wu et al., 2024).

#### 3.2.3 Additional HDAC modulators

Baicalein from *Scutellaria baicalensis* demonstrates both HDAC and SIRT1 modulation in preclinical diabetes studies. This flavonoid compound promotes β-cell survival through anti-apoptotic effects while improving glucose metabolism through chromatin remodeling (Szkudelski and Szkudelska, 2023; Baradaran Rahimi et al., 2021; Yingrui et al., 2022). Luteolin from Chrysanthemum species shows HDAC and HAT modulation activity in preclinical diabetes models. This compound provides pancreatic protection through chromatin remodeling while demonstrating direct metabolic benefits (Alam et al., 2015; Bhattacharjee and Dashwood, 2020; Rajaselvi et al., 2023).

### 3.3 Dual-target natural products: comprehensive epigenetic modulation

#### 3.3.1 Genistein: isoflavone with broad epigenetic effects

Genistein from *Glycine max* (soybean) represents a particularly promising dual-target compound that modulates both HDAC and DNMT activities. This isoflavone has progressed to Phase II clinical trials for metabolic syndrome and type 2 diabetes, demonstrating remarkable effects on islet regeneration and glucose homeostasis (Kochar Kaur, 2021; (Ramírez-Alarcón et al., 2021; Han et al., 2023). The mechanism of genistein involves simultaneous chromatin remodeling through HDAC inhibition and DNA demethylation through DNMT modulation. This dual action results in comprehensive reactivation of silenced metabolic genes while promoting β-cell regeneration and improved insulin sensitivity. Clinical studies have validated genistein’s therapeutic potential for diabetes prevention and treatment.

#### 3.3.2 Sulforaphane: multi-target epigenetic modulator

Sulforaphane demonstrates the most comprehensive epigenetic modulation profile among natural products, inhibiting both HDAC and DNMT activities while activating beneficial transcriptional pathways. Beyond its DNMT1 inhibitory effects discussed earlier, sulforaphane also modulates HDAC activity, creating synergistic effects on gene expression. The compound’s progression to Phase I clinical trials validates its therapeutic potential for diabetes treatment (Gupta et al., 2022; Andrés et al., 2025).

#### 3.3.3 Additional multi-target compounds

Apigenin from *Petroselinum crispum* (parsley) demonstrates both HDAC and HAT modulation in preclinical diabetes studies. This flavonoid improves β-cell function through chromatin remodeling while providing insulin resistance benefits (Ayipo et al., 2024; Pandey et al., 2012; Cheng et al., 2021; Pandey et al., 2012). Withaferin A from *Withania somnifera* combines HDAC inhibition with NF-κB suppression, addressing both epigenetic dysfunction and inflammatory processes in diabetes. Preclinical studies demonstrate significant anti-diabetic effects through this dual mechanism (Kumar et al., 2023; Nisar et al., 2025; Saha et al., 2024).

### 3.4 Mechanistic integration and therapeutic implications

The natural products examined above demonstrate varying degrees of selectivity and potency against DNMT1 and HDAC3, the key epigenetic regulators identified in diabetes pathogenesis. Compounds like berberine and curcumin have achieved clinical validation through Phase II-III trials, confirming that natural products can achieve therapeutically relevant epigenetic modulation in human subjects (Dai et al., 2024). The progression from preclinical to clinical development reveals important patterns in natural product therapeutic development. Compounds with established traditional use and favorable safety profiles, such as curcumin and berberine, have advanced more rapidly through clinical development compared to more recently discovered compounds (Othman et al., 2025).

The dual-target compounds represent particularly interesting therapeutic opportunities, as they address the complex nature of epigenetic dysfunction in diabetes. Rather than requiring combination therapy with multiple agents, single compounds like genistein and sulforaphane can simultaneously reverse both DNA methylation and histone deacetylation abnormalities (Paul et al., 2018).

## 4 Carbohydrate-metabolizing enzymes as therapeutic targets

Post meal glucose spikes represent a defining characteristic of diabetes that significantly drives disease worsening and long-term complications. The enzymatic conversion of dietary starches to glucose creates several opportunities for therapeutic intervention (Hanssen et al., 2020). This digestive process starts with salivary α-amylase working in the mouth to begin breaking down complex carbohydrates. Following temporary inactivation in the acidic stomach environment, pancreatic α-amylase resumes the breakdown process within the small intestine, transforming starches into intermediate sugars like maltose and maltotriose (Peyrot des Ga et al., 2016). The concluding step takes place at the intestinal brush border, where α-glucosidase enzymes split these smaller sugar chains into individual glucose molecules ready for absorption into the bloodstream (Date, 2021). Figure 3 demonstrates this complete anatomical journey from dietary starch consumption to glucose uptake, clearly showing the location and function of each critical enzyme along the digestive pathway.

**FIGURE 3:**
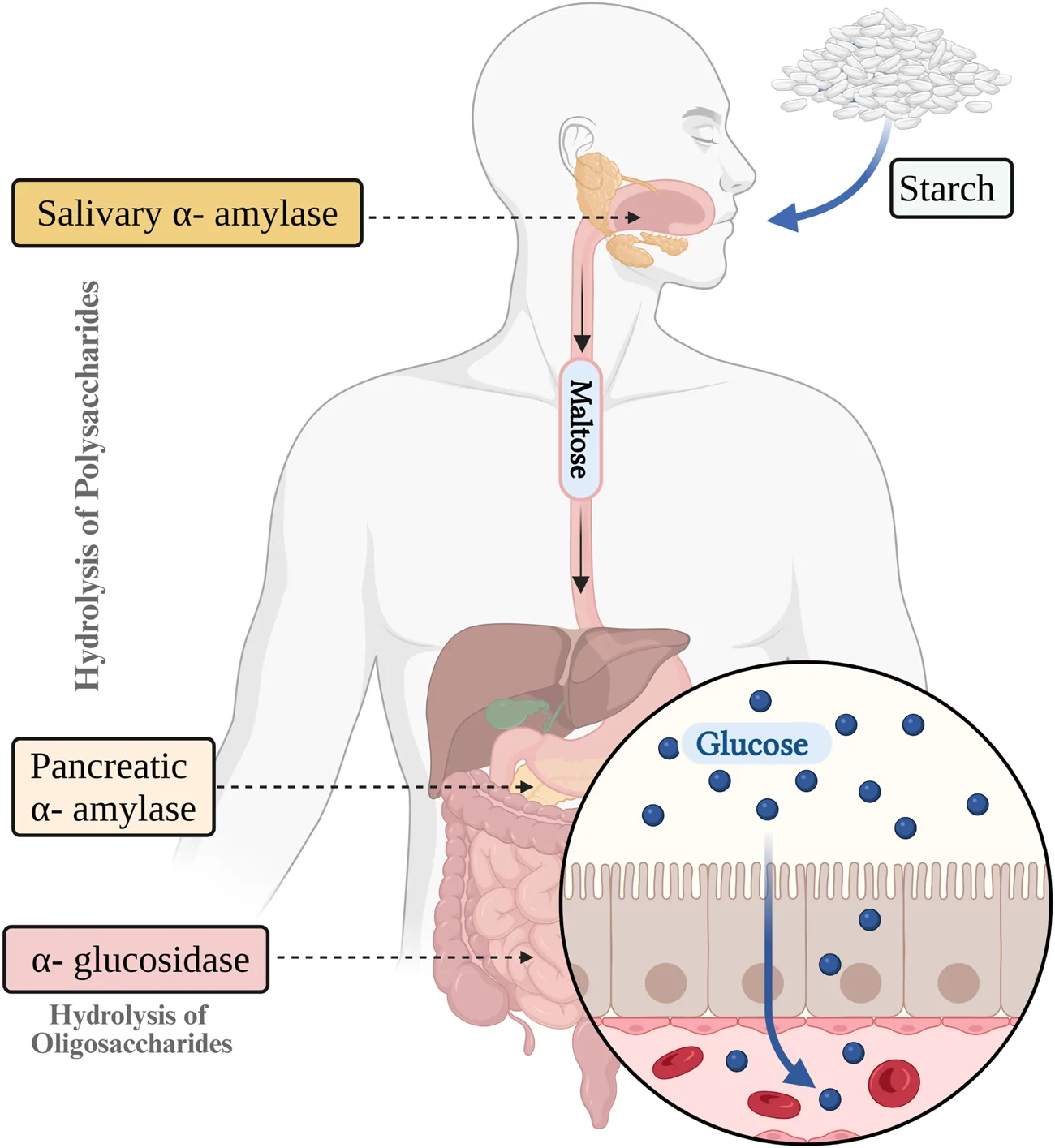
Carbohydrate Digestion Pathway and Enzyme Targets. Anatomical representation showing the sequential breakdown of dietary starch through three key enzymes: salivary α-amylase in the oral cavity initiating polysaccharide hydrolysis, pancreatic α-amylase in the small intestine completing starch conversion to oligosaccharides, and α-glucosidase at the intestinal brush border performing final glucose liberation. The pathway illustrates therapeutic intervention points where enzyme inhibitors can control postprandial glucose absorption. Created with BioRender.com, accessed on 14 June 2025.

Both enzyme families have demonstrated clinical value as therapeutic targets for controlling after-meal glucose elevations (Alqahtani et al., 2019). Nevertheless, completely blocking α-amylase activity can trigger digestive problems from bacterial fermentation of unprocessed starches in the colon, making moderate inhibition the preferred approach (Kashtoh and Baek, 2023). α-Glucosidase presents an especially attractive target because of its position at the intestinal wall where final glucose release occurs, allowing inhibitors like acarbose to work locally with reduced whole-body effects (Harsch and Konturek, 2018). Various natural and synthetic compounds that target these enzymes employ either direct active-site binding or indirect allosteric mechanisms, providing possibilities for effective glucose management with fewer adverse effects (Kalinovskii et al., 2023).

### 4.1 Natural product inhibitors: chemical diversity and therapeutic applications

The landscape of natural α-amylase and α-glucosidase inhibitors encompasses remarkable structural diversity, ranging from simple phenolic compounds to complex polycyclic molecules derived from various plant families (Ćorković et al., 2022). Search Methodology: Natural α-amylase and α-glucosidase inhibitors were systematically compiled through comprehensive literature analysis spanning 2010–2025. Database searches across PubMed, Scopus, and Science Direct combined terms “α-glucosidase inhibitors” and “α-amylase inhibitors” with “natural products,” “phytochemicals,” and specific chemical classes. From extensive literature reporting, we curated 100 natural compounds prioritizing: (1) documented enzyme inhibitory activity regardless of potency range to capture the full spectrum of natural modulators, (2) experimental validation with reproducible methodologies, (3) structural diversity representing major phytochemical families, and (4) authenticated botanical sources. While IC_50_ values varied considerably—from sub-micromolar to millimolar concentrations—this range reflects the natural variation in plant-derived enzyme modulators and their potential for optimization. Data harmonization required converting diverse units (μM, μg/mL, mg/mL, mM) reported across studies, with information verified through original research articles and corroborating reviews where available. Table 1 presents this comprehensive collection, including compounds across all potency ranges, as even moderate inhibitors may possess valuable structural features for cross-target activity or serve as scaffolds for future optimization.

**TABLE 1:** α-Glucosidase and α-Amylase inhibitors from natural products.

Sr No	Compound	Chemical class	Plant source	α-Glucosidase IC_50_ (μM)	α-Amylase IC_50_ (μM)	Inhibition type	Clinical status	Original reference
1	Acarbose (Standard)	Oligosaccharide	*Actinoplanes sp.*	0.62 ± 0.07	1.25 ± 0.15	Competitive	FDA Approved	Tuyen et al. (2021)
2	Voglibose	N-substituted valiolamine	Synthetic derivative	68.12 ± 0.04	46.5 ± 2.8	Competitive	FDA Approved	Karthikeyan et al. (2021)
3	Miglitol	Iminosugar	Synthetic derivative	11.47 ± 0.02	45.2 ± 3.5	Competitive	FDA Approved	Sheikh et al. (2019)
4	Procyanidin A2	Proanthocyanidin	*Vitis vinifera*	0.27 ± 0.01 μg/mL	14.08 ± 1.2 μg/mL	Non-competitive	Research stage	Sheikh et al. (2019)
5	Salacinol	Thiosugar sulfonium	*Salacia reticulata*	3.2 ± 0.15	8.5 ± 0.42	Competitive	Traditional Medicine	Yoshikawa et al. (2002)
6	Morusin	Prenylated flavonoid	*Morus alba*	3.19 ± 0.21	12.4 ± 0.85	Mixed	Research stage	Chen et al. (2018)
7	Corilagin	Ellagitannin	*Phyllanthus amarus*	1.70 ± 0.03	28.4 ± 1.2	Mixed	Research stage	Trinh et al. (2016)
8	Repandusinic acid A	Ellagitannin	*Phyllanthus urinaria*	6.10 ± 0.10	35.2 ± 2.1	Competitive	Research stage	
9	Mallotinin	Ellagitannin	*Phyllanthus urinaria*	3.76 ± 0.15	42.1 ± 2.8	Competitive	Research stage	
10	Kotalanol	Thiosugar sulfonium	*Salacia reticulata*	4.1 ± 0.18	9.2 ± 0.56	Competitive	Traditional Medicine	Muraoka et al. (2010)
11	EGCG	Gallate ester	*Camellia sinensis*	4.03 ± 0.01	45.30 ± 0.22	Non-competitive	GRAS status	Wu et al. (2019)
12	Taxumariene F	Taxane derivative	*Taxus wallichiana*	3.7 ± 0.75	18.6 ± 1.3	Competitive	Research stage	Chen et al. (2020)
13	Chrotacumine C	Alkaloid	*NPASS Database*	8.42	14.46	Mixed	*In silico* validated	Ndarawit et al. (2024)
14	Rhaponticin	Stilbene glycoside	*Rheum tanguticum*	0.3 ± 1.6	48.6 ± 3.2	Competitive	Research stage	Yang et al. (2017)
15	Candidone	Flavonoid	*Tephrosia candida*	1.40 ± 0.20	7.46 ± 0.73	Not specified	Research stage	Iran et al. (2025)
16	Quercetin	Flavonol	*Allium cepa*	1.68 ± 0.04	1.15 ± 0.03	Non-competitive	GRAS status	Oboh et al. (2015a)
17	Isoliquiritigenin styrylchromone)	Flavonoid	*Edgeworthia gardneri*	22.3 ± 0.8	Not reported	Mixed	Research stage	Li et al. (2024)
18	Tiliroside	Flavonoid	*E. gardneri*	23.6 ± 0.3	Not reported	Mixed	Research stage	
19	Luteolin	Flavone	*Reseda luteola*	104.8 ± 1.8	38.5 ± 2.5	Mixed	Research stage	Xu et al. (2024a)
20	Ursolic acid	Triterpenoid	*Rosmarinus officinalis*	5.08 ± 0.70	35.8 ± 2.4	Competitive	Research stage	Wu et al. (2017)
21	Epicatechin gallate (ECG)	Gallate ester	*Camellia sinensis*	27 ± 1.9	178.4 ± 12.5	Mixed	GRAS status	Kamiyama et al. (2010)
22	Betulinic acid	Triterpenoid	*Betula spp.*	7.21 ± 0.58	48.9 ± 3.3	Competitive	Research stage	Wu et al. (2023)
23	Mulberrofuran K	Prenylated flavonoid	*Morus alba*	2.29**–**5.91	45.3 ± 3.2	Mixed	Research stage	Ha et al. (2018)
24	Mulberrofuran G	Prenylated flavonoid	*Morus alba*	2.29–5.91	32.6 ± 2.3	Mixed	Research stage	
25	Akebonoic acid	Triterpenoid	*Akebia quinata*	0.009 ± 0.001	38.2 ± 2.5	Competitive	Research stage	Ouyang et al. (2018)
26	Rosiridin	Monoterpene	*Rhodiola rosea*	40.62 ± 3.74	51.97 ± 6.32	Not specified	Research stage	Iran et al. (2025)
27	Carnosic acid	Diterpene	*Rosmarinus officinalis*	16.70 μg mL^–1^	1.56 μg mL^–1^	Mixed	Research stage	Sheng et al. (2018)
28	Curcumin	Curcuminoid	*Curcuma longa*	28.5 ± 1.6	21.0 ± 3.2	Mixed	Clinical trials	Roney et al. (2024)
29	Berberine	Isoquinoline alkaloid	*Berberis spp.*	198.4 μg mL^–1^	50.83 μg mL^–1^	Mixed	Clinical trials	Zhao et al. (2022)
30	Isoorientin	Flavone C-glycoside	*Passiflora spp.*	38.2 ± 2.6	58.3 ± 4.0	Mixed	Research stage	Şöhretoğlu and Sari (2020)
31	Oleanolic acid	Triterpenoid	*Olea europaea*	6.35 ± 0.02	62.5 ± 4.2	Non Competitive	Research stage	Ding et al. (2018)
32	Orientin	Flavone C-glycoside	*Passiflora spp.*	0.13 mg/mL	>270 μg/mL	Mixed	Research stage	(Yang et al., 2014; Sadeghi et al., 2023)
33	Baicalein	Flavone	*Scutellaria baicalensis*	59.32	179.1	Mixed	Traditional Medicine	Dinesh et al. (2022)
34	Mangiferin	Xanthone glycoside	*Mangifera indica*	36.84 μg/mL	63.57 μg/mL	Non-competitive	Traditional Medicine	Sekar et al. (2019)
35	2,3-Epoxyprocyanidin C1	Proanthocyanidin	*Pterocarpus erinaceus*	41.6 ± 1.0 μg/mL	>50 μg/mL	Mixed	Research stage	Feunaing et al. (2022)
36	Adicardin	Coumarin Glycoside	*Gmelina arborea*	20.18 ± 1.58	24.92 ± 3.01	Not specified	Research stage	Iran et al. (2025)
37	Astragalin	Flavonol glycoside	*Astragalus spp.*	154.5	68.2 ± 4.5	Competitive	Research stage	Li et al. (2025)
38	Corymbiferin	Coumarin	*Ferulago angulata*	48.5 ± 3.1	72.3 ± s4.8	Mixed	Research stage	Gupta et al. (2021)
39	Rutin	Flavonoid glycoside	*Fagopyrum esculentum*	0.043	0.037	Competitive	GRAS status	Oboh et al. (2015a)
40	Kaempferol	Flavonol	*Kaempferia galanga*	52.95 μg mL^−1^	2.33 μg mL^−1^	Mixed	Research stage	Sheng et al. (2018)
41	Theaflavin	Flavanol dimer	*Fermented tea*	16.17	0.46	Mixed	Research stage	Lam et al. (2023)
42	Myricetin	Flavonol	*Myrica spp.*	1.030 ± 0.026	<1.030 ± 0.026	Mixed	Research stage	Ng (2016)
43	Naringin	Flavanone glycoside	*Citrus spp.*	0.55 ± 0.3	8.0 ± 0.1	Competitive	GRAS status	Sahnoun et al. (2017)
44	Apigenin	Flavone	*Petroselinum crispum*	10.5 ± 0.05	52.3 ± 3.5	Competitive	Research stage	Zeng et al. (2016)
45	Cyanidin-3-O-glucoside	Anthocyanin	*Vaccinium spp.*	13.72	7.5	Mixed	GRAS status	Choi et al. (2017)
46	Comselogoside	Secoiridoid glycoside	*Olea europaea*	655 ± 36 μg mL^−1^	338.2 ± 34.4 μg mL^−1^	Mixed	Research stage	Rubio‐Senent et al. (2025)
47	Delphinidin-3-O-glucoside	Anthocyanin	*Vaccinium spp.*	19.7 ± 0.24	105.8 ± 7.1	Mixed	GRAS status	Cásedas et al. (2019)
48	3,4-Dihydroxyphenylglycol (DHPG)	Phenylethanol derivative	*Olea europaea*	48.9 ± 1.8 μg mL^−1^	70.13 ± 2.60 μg mL^−1^	Mixed	Research stage	Rubio‐Senent et al. (2025)
49	Gymnemic acid I	Triterpenoid saponin	*Gymnema sylvestre*	65.77 ± 5.1	68.12 ± 6.3	Mixed	Traditional Medicine	Karthikeyan et al. (2021)
50	katononic acid	Diterpenoid acid	*Nuxia oppositifolia*	194.8	119.3	Mixed	GRAS status	Alqahtani et al. (2019)
51	Isorhamnetin	Flavonol	*Hippophae rhamnoides*	19.68	29.9 μg mL^−1^	Mixed	Research stage	(Yang et al., 2014; Li et al., 2009)
52	Protocatechuic acid	Phenolic acid	*Hibiscus sabdariffa*	0.90 ± 0.01 mM	2.65 ± 0.16 mM	Non-competitive	GRAS status	Aleixandre et al. (2022)
53	Hyperoside	Flavonol glycoside	*Hypericum perforatum*	No	0.491 μg mL^−1^	Mixed	Research stage	Shen et al. (2023)
54	Catechin	Flavan-3-ol	*Camellia sinensis*	∼31 μg mL^−1^	160 ± 67 μg mL^−1^	Competitive	GRAS status	Yilmazer-Musa et al. (2012)
55	Glabridin	Isoflavane	*Glycyrrhiza glabra*	134.2–164.5 μg/mL	236.2 μg/mL	Mixed	Research stage	Paul and Raza (2024)
56	Quercitrin	Flavonol glycoside	*Quercus spp.*	0.038	0.061	Competitive	Research stage	Oboh et al. (2015a)
57	Epicatechin	Flavan-3-ol	*Camellia sinensis*	92.3 ± 6.1	512.0 ± 34.1	Competitive	GRAS status	Wu et al. (2019)
58	Chicoric acid	Phenolic acid	*Cichorium intybus*	0.28 mg/mL	12.29 – 0.03 mg/mL	Mixed	Research stage	Chiou et al. (2017)
59	Genistein	Isoflavone	*Glycine max*	0.11 ± 0.04 mg/mL	0.69 ± 0.06 mg/mL	Mixed	Dietary supplement	Jia et al. (2024)
60	Sinapic acid	Phenolic acid	*Brassica spp.*	1.48 ± 0.08 mM	>8.92 mM	Mixed	Research stage	Aleixandre et al. (2022)
61	3-Oxolupenal	Triterpenoid	*Nuxia oppositifolia*	141.8 ± 9.4	101.6 ± 6.8	Mixed	Research stage	Alqahtani et al. (2019)
62	p-Coumaric acid	Phenolic acid	*Solanum lycopersicum*	6.20 ± 0.04 mM	52.48 ± 0.89 mM	Competitive	Research stage	Aleixandre et al. (2022)
63	Eriocitrin	Flavanone glycoside	*Citrus spp.*	12.50 ± 0.73	145.8 ± 9.7	Mixed	GRAS status	Sadeghi et al. (2022)
64	Daidzein	Isoflavone	*Glycine max*	0.048 mM	0.301 mM	Mixed	Dietary supplement	Park et al. (2013)
65	Liquiritigenin	Flavanone	*Glycyrrhiza spp.*	27.91 ± 3.48	178.5 ± 11.9	Mixed	Research stage	Zhou et al. (2025)
66	Phlorizin	Dihydrochalcone glycoside	*Malus domestica*	0.97 mM	145.6 ± 9.7	Competitive	Research stage	Xu et al. (2024b)
67	Diosmetin	Flavone	*Citrus spp.*	18.3 ±1.26	155.2 ± 10.3	Mixed	Research stage	Zhang et al. (2024)
68	Naringin	Flavanone glycoside	*Citrus spp.*	0.55	8	Mixed	GRAS status	Sahnoun et al. (2017)
69	Isoliquiritigenin	Chalcone	*Glycyrrhiza spp.*	125.8 ± 8.3	158.2 ± 10.5	Mixed	Research stage	Wei et al. (2024)
70	Rosmarinic acid	Phenolic acid	*Rosmarinus officinalis*	0.34 mg/mL	Not reported	Mixed	GRAS status	XN et al. (2018)
71	β-Sitosterol	Phytosterol	*Multiple plants*	54.5	216.02	Non-competitive	Dietary supplement	Nkobole et al. (2011)
72	Narirutin	Flavanone glycoside	*Citrus spp.*	0.00091 mg/mL	1.012 mg/mL	Mixed	GRAS status	Qurtam et al. (2021)
73	Poncirin	Flavanone glycoside	*Poncirus trifoliata*	125.5 ± 0.2	1.0 ± 0.2	Competitive	Research stage	Sahnoun et al. (2017)
74	Formononetin	Isoflavone	*Trifolium pratense*	0.027 mM	Not reported	Mixed	Research stage	Jiang et al. (2015)
75	Katonic acid	Triterpenoid	*Nuxia oppositifolia*	194.8 ± 12.9	119.3 ± 7.9	Mixed	Research stage	Alqahtani et al. (2019)
76	Nobiletin	Polymethoxylated flavone	*Citrus depress*	4.2 ± 0.3	50 ± 0.3	Competitive	Research stage	Sahnoun et al. (2017)
77	Didymin	Flavanone glycoside	*Poncirus* *trifoliata*	4.20 ± 0.6	<4.20 ± 0.6	Mixed	Research stage	Tundis et al. (2016)
78	Hesperidin	Flavanone glycoside	*Citrus spp.*	111 ± 0.3	6 ± 0.2	Mixed	GRAS status	Sahnoun et al. (2017)
79	Hydroxytyrosol (HT)	Phenolic alcohol	*Olea europaea*	192 ± 5 μg/mL	171.7 ± 39.5 μg/mL	Competitive	Research stage	Rubio‐Senent et al. (2025)
80	Betulinic acid	Triterpenoid	*Betula spp.*	0.27 μg/mL	70.02 μg/mL	Mixed	Research stage	Salau et al. (2024)
81	Tangeretin	Polymethoxylated flavone	*Citrus reticulata (peel)*	141 ± 0.2	14.8 ± 0.1	Competitive	Research stage	Sahnoun et al. (2017)
82	Rhoifolin	Flavone glycoside	*Citrus spp.*	0.54 mg/mL	0.58 mg/mL	Mixed	Research stage	Ehsan et al. (2025)
83	Protocatechuic acid	Phenolic acid	*Multiple plants*	1.76 μg/mL	1.12 μg/mL	Mixed	GRAS status	Adefegha et al. (2015)
84	Caffeic acid	Phenolic acid	*Multiple plants*	4.98 μg/mL	3.68 μg/mL	Mixed	GRAS status	Oboh et al. (2015b)
85	Eriodictyol	Flavanone	*Andromachia igniaria*	0.02 mM	0.07 mM	Mixed	Research stage	Saltos et al. (2015)
86	Vanillic acid	Phenolic acid	*Oryza sativa*	0.100 ± 0.01 mmol L^−1^	0.130 ± 0.02 mmol L^−1^	Mixed	Research stage	Sansenya and Payaka (2022)
87	Galangin	Flavonol	*Alpinia galanga*	63.86 μg mL^−1^	4.52 μg mL^−1^	Mixed	Research stage	Sheng et al. (2018)
88	Oxyresveratrol	Stilbene	*Morus alba*	203.7 ± 4.6	Not siginificant	Mixed	Research stage	Wongon and Limpeanchob (2020)
89	Chrysin	Flavone	*Passiflora spp.*	182.5 ± 12.1	225.6 ± 15.0	Competitive	Research stage	Khali et al. (2022)
90	Ellagic acid	Polyphenol	*Multiple plants*	3.65 ± 0.09 μg/mL	>3.65 ± 0.09 μg/mL	Mixed	GRAS status	Yin et al. (2018)
91	Chlorogenic acid	Phenolic acid	*Coffee spp.*	9.24 μg/mL	9.10 μg/mL	Competitive	GRAS status	Oboh et al. (2015b)
92	Fisetin	Flavonol	*Rhus spp.*	32.77	158.2 ± 10.5	Mixed	Research stage	Yue et al. (2018)
93	Xanthohumol	Flavonoid	*Humulus lupulus*	32.58 ± 3.11	71.07 ± 5.82	Mixed	Research stage	Zhang et al. (2025)
94	Gallic acid	Phenolic acid	*Multiple plants*	1.22 μg/mL	1.09 μg/mL	Competitive	GRAS status	Adefegha et al. (2015)
95	Syringic acid	Phenolic acid	*Multiple plants*	8.25 ± 0.09 mM	1.98 ± 0.01 mM	Mixed	Research stage	Aleixandre et al. (2022)
96	Piceatannol	Stilbene	*Vitis vinifera*	20.4 ± 7.6 μg/mL	85.4 ± 0.7 μg/mL	Mixed	Research stage	dos Santos et al. (2022)
97	Schaftoside	Flavone C-glycoside	*Desmodium spp.*	0.078 ± 0.06 mM	0.3067 ± 0.35 mM	Mixed	Research stage	Kan et al. (2023)
98	Ferulic acid	Phenolic acid	*Multiple plants*	0.866 mg mL^−1^	0.622 mg mL^−1^	Competitive	GRAS status	Zheng et al. (2020)
99	Isovitexin	Flavone C-glycoside	*Vitex spp.*	266.2	328.5	Mixed	Research stage	Choo et al. (2012)
100	Vitexin	Flavone C-glycoside	*Ficus deltoidea*	244.0	315.0	Mixed	Research stage	

IC_50_ values are reported in various units as published in the original literature. Some compounds data is not reported.

The selected compounds demonstrate extraordinary chemical diversity spanning multiple major phytochemical classes, with flavonoids representing the largest group, followed by terpenoids, phenolic acids, alkaloids, and other specialized metabolites including stilbenes, coumarins, and anthraquinones. This structural variety reflects different evolutionary strategies that plants have developed to modulate carbohydrate metabolism, creating a rich source of potential therapeutic agents with diverse binding modes and selectivity profiles. The extensive characterization of these compounds’ enzyme inhibition mechanisms, safety profiles, and pharmacokinetic properties provides an ideal foundation for cross-target repurposing studies (Chaa et al., 2024). Figure 4 illustrates the chemical classification distribution of these 100 selected inhibitors, highlighting the predominance of polyphenolic structures that typically achieve enzyme inhibition through hydrogen bonding interactions with active site residues, while simultaneously possessing the structural features commonly associated with epigenetic modulator compounds.

**FIGURE 4:**
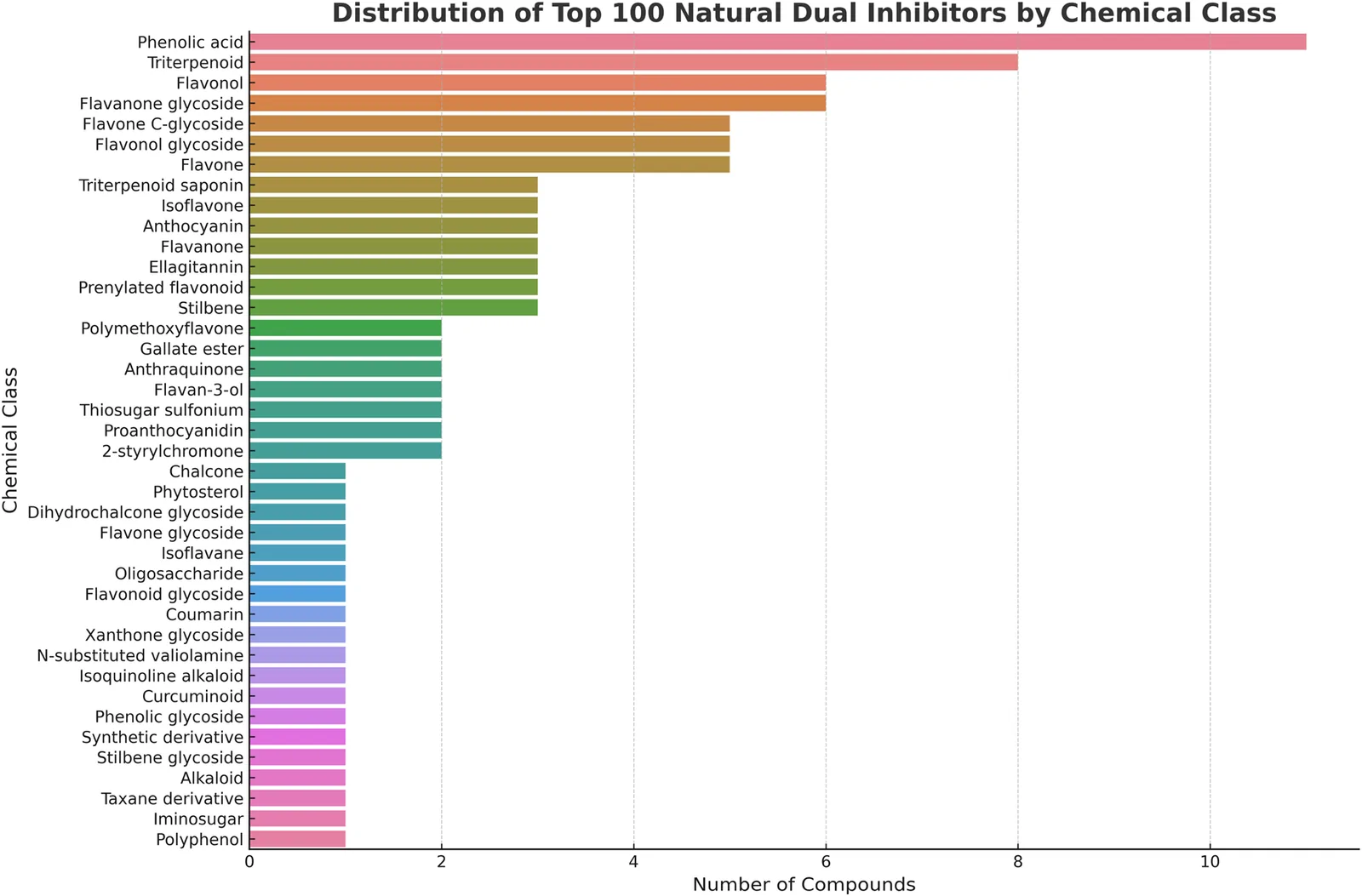
Chemical Classification of Natural α-Amylase and α-Glucosidase Inhibitors. The pie chart shows the distribution of 100 systematically selected natural product inhibitors across major phytochemical classes. Flavonoids constitute the largest group (48 compounds, 48%), followed by terpenoids including monoterpenes, diterpenes and triterpenoids (15 compounds, 15%), phenolic acids (13 compounds, 13%), specialized glycosides and other metabolites (18 compounds, 18%) including oligosaccharides, thiosugars, and secoiridoids, with smaller contributions from ellagitannins, stilbenes, coumarins, and alkaloids (6 compounds, 6%). The predominance of polyphenolic structures (flavonoids + phenolic acids = 61%) aligns with known structural requirements for both enzyme inhibition and epigenetic modulation activities.

Berberine exemplifies the most clinically advanced natural enzyme inhibitor, demonstrating potent activity against both α-amylase and α-glucosidase through mixed inhibition mechanisms. Clinical trials have validated berberine’s glucose-lowering efficacy, with effects comparable to conventional antidiabetic medications. The compound’s dual enzymatic activity, combined with additional metabolic benefits, positions it as a promising alternative to synthetic inhibitors (Yin et al., 2008).

Flavonoid compounds constitute the largest class of natural enzyme inhibitors, with quercetin, curcumin, and catechins showing particularly strong inhibitory potential. These polyphenolic structures typically achieve inhibition through hydrogen bonding interactions with active site residues, creating stable enzyme-inhibitor complexes that prevent normal substrate processing. Many flavonoids possess Generally Recognized as Safe (GRAS) status, facilitating their clinical development and therapeutic application (Panche et al., 2016).

Tea catechins, including epicatechin, catechin, and epigallocatechin gallate (EGCG), demonstrate selective α-glucosidase inhibition with competitive binding mechanisms. Their widespread consumption through tea intake provides population-level evidence for safety and tolerability, while controlled studies confirm their glucose-lowering potential in diabetic individuals (Singh et al., 2011).

Phenolic acids and their derivatives represent another important class of natural inhibitors, with compounds like chlorogenic acid and caffeic acid derivatives showing moderate to strong enzymatic inhibition. These compounds often occur in commonly consumed foods, suggesting potential for dietary interventions that complement pharmacological approaches (Kumar and Goel, 2019).

### 4.2 Structure-activity relationships and mechanistic insights

Analysis of natural enzyme inhibitors reveals important structural determinants that govern inhibitory potency and selectivity. Flavonoid inhibitors typically require specific hydroxylation patterns for optimal binding, with the presence and positioning of hydroxyl groups significantly influencing inhibitory activity. The addition of gallate esters, as observed in EGCG, often enhances binding affinity through additional hydrogen bonding interactions (Falcone Ferreyra et al., 2012).

Ring substitution patterns also influence inhibitory mechanisms, with certain structural features favoring competitive versus non-competitive binding modes. Planar aromatic systems facilitate π-π stacking interactions with aromatic amino acids in enzyme active sites, while flexible substituents may access allosteric binding sites that modulate enzyme conformation (Saroha et al., 2025).

The relationship between chemical structure and inhibition type has important therapeutic implications. Competitive inhibitors may require higher concentrations to achieve sustained inhibition under physiological substrate concentrations, while non-competitive inhibitors can maintain their effects regardless of substrate availability. Mixed inhibition patterns, observed with compounds like berberine and curcumin, may provide optimal therapeutic profiles by combining both binding modes (Pelley, 2012).

## 5 Therapeutic repurposing: unlocking dual-target potential

Despite extensive characterization of metabolic enzyme inhibitors for diabetes, their epigenetic activities remain unexplored. Among our systematically selected 100 compounds, several have already been reported to possess both metabolic enzyme inhibition and epigenetic modulatory activities in separate research contexts. Berberine demonstrates potent α-glucosidase inhibition while also functioning as a DNMT1 inhibitor in cancer studies. Curcumin shows α-amylase inhibitory activity alongside established HDAC inhibition properties (McCubrey et al., 2017). EGCG exhibits dual α-glucosidase inhibition and DNMT1 modulation, yet these dual activities have never been systematically investigated for insulin resistance treatment (Agarwal et al., 2023; Wu et al., 2019). These serendipitous discoveries suggest that many other metabolic enzyme inhibitors may possess unrecognized epigenetic activities.

The biological rationale for this cross-target potential lies in the interconnected nature of metabolic and epigenetic regulatory systems. Figure 5 illustrates the molecular crosstalk between these pathways, revealing multiple points where therapeutic intervention could simultaneously address both metabolic enzyme dysfunction and epigenetic dysregulation.

**FIGURE 5:**
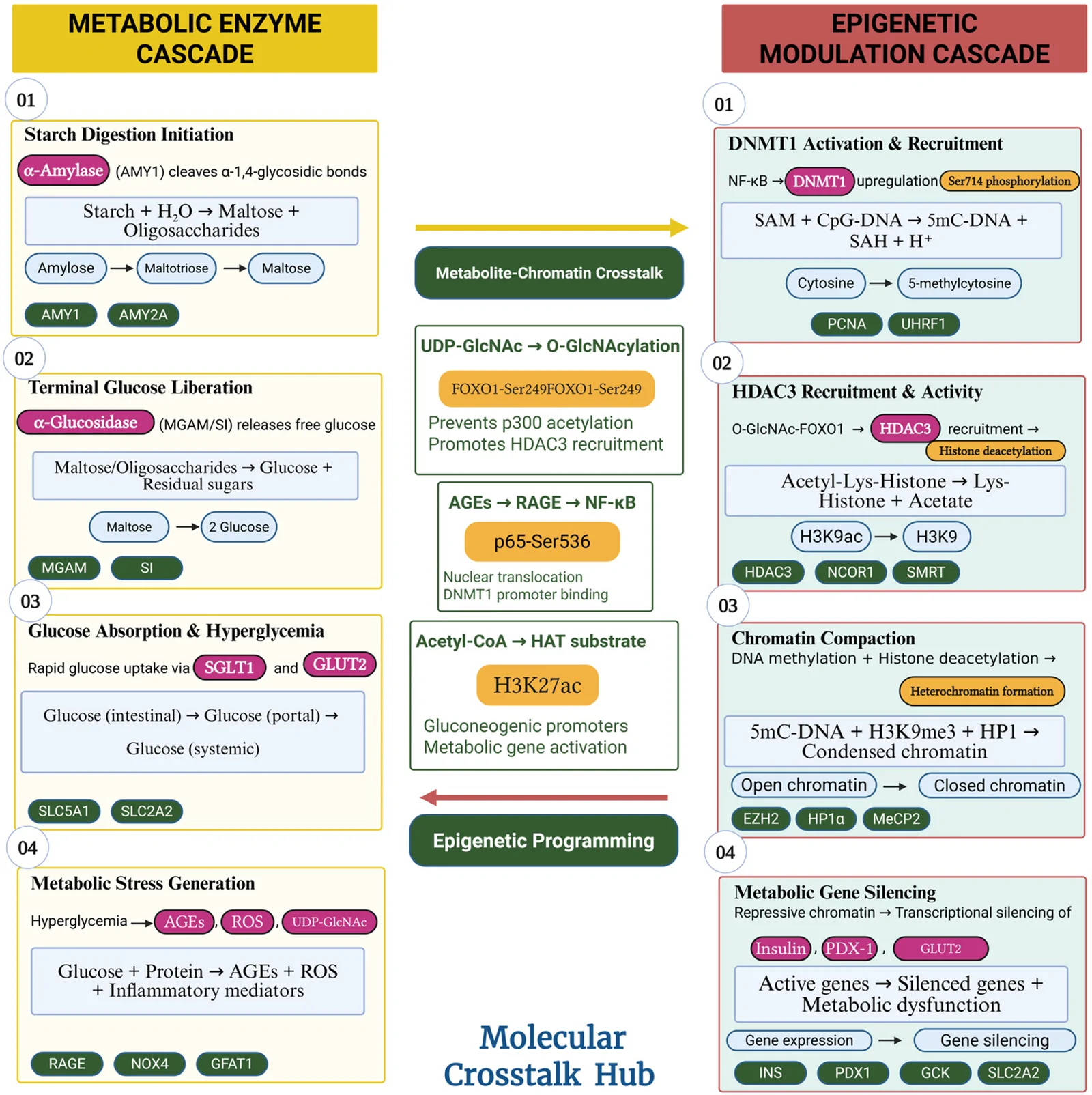
Metabolic-Epigenetic Pathway Crosstalk in Insulin Resistance. Schematic diagram showing the bidirectional communication between metabolic enzyme pathways and epigenetic regulatory networks. Hyperglycemia from uncontrolled carbohydrate digestion activates DNMT1 and HDAC3, which silence insulin signalling genes, creating a self-perpetuating cycle. The diagram highlights intervention points where dual-target compounds could simultaneously inhibit glucose absorption (via metabolic enzymes) and restore gene expression (via epigenetic modulators), breaking the pathological cycle at multiple levels (Kaimala et al., 2023; (Kiełbowski et al., 2025; Caturano et al., 2023; Ning et al., 2021; Gilbert and Liu, 2012; Colagiuri and Ceriello, 2025). Created with BioRender.com, accessed on 15 June 2025.

### 5.1 Structural foundation for cross-target activity

The molecular basis for cross-target activity becomes evident when examining the structural features shared between metabolic and epigenetic enzymes (See Table 2) Both enzyme families utilize similar cofactor requirements, binding pocket architectures, and catalytic mechanisms that create opportunities for small molecule cross-reactivity (Janeček et al., 2014; Curci et al., 2024). Figure 6 demonstrates these structural conservation patterns that enable natural products to achieve dual-target activities.

**TABLE 2:** Structural comparison of binding pockets among metabolic (α-amylase, α-glucosidase) and epigenetic enzymes (HDAC3, DNMT1).

Feature	α-Amylase	α-Glucosidase	HDAC3	DNMT1
Pocket Volume (Å^3^)	18.2	16.7	17.5	15.9
Aromatic Residues	4–5	3–4	4–5	3–4
H-bond Acceptors	6–8	5–7	6–7	5–6
Metal Coordination	Yes	No	Yes	No
Hydrophobic Regions	65%	70%	60%	68%
Pocket Depth (Å)	14.2	12.8	15.1	13.6
Secondary Structure Composition	Mixed (α/β) barrel	Mixed (β/α)_8_ fold	α-helical bundle	β-sheet core with loops

**FIGURE 6:**
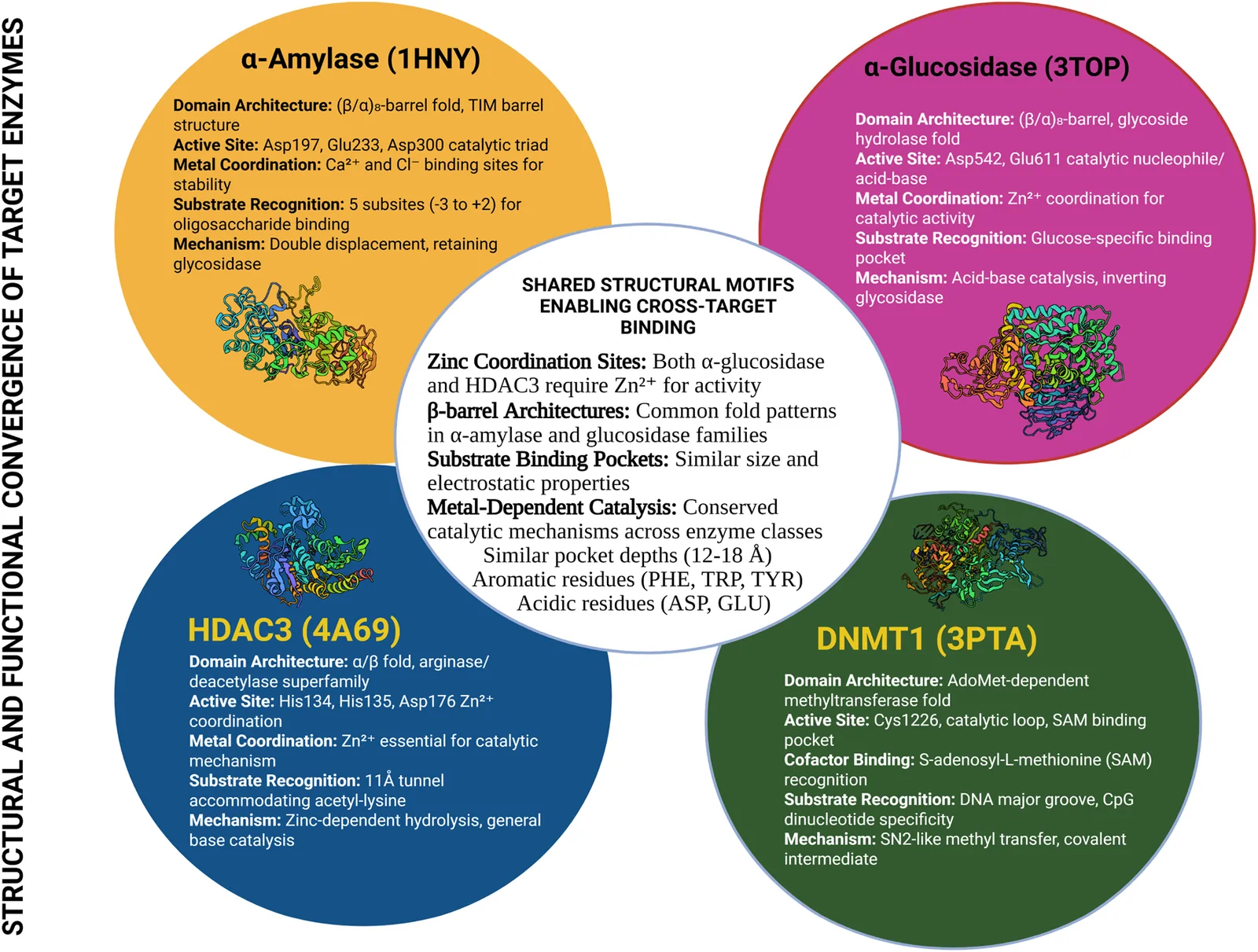
Structural Conservation Between Metabolic and Epigenetic Enzyme Families. Comparative analysis of active site structures showing conserved binding features between α-amylase/α-glucosidase and DNMT1/HDAC3. Key structural similarities include zinc coordination sites, aromatic binding pockets, and hydrophobic regions that accommodate natural product inhibitors. The structural overlay reveals how compounds with appropriate pharmacophores can potentially bind to multiple targets, providing the molecular foundation for cross-target therapeutic activity (Neves et al., 2022; Alkaff et al., 2021). Created with BioRender.com, accessed on 16 June 2025.

Natural products have evolved to interact with multiple biological targets simultaneously, creating inherent polypharmacology that modern drug development often eliminates through selectivity optimization (Choo and Chai, 2023). However, for complex diseases like insulin resistance involving multiple dysregulated pathways, this natural promiscuity represents a therapeutic advantage. The structural similarities between enzyme families provide the molecular foundation enabling this cross-target potential.

### 5.2 Therapeutic rationale for integrated targeting

The temporal relationship between metabolic dysfunction and epigenetic silencing supports an integrated therapeutic approach. Post-meal glucose spikes from inadequate carbohydrate enzyme inhibition directly trigger DNMT1 and HDAC3 activation within hours, initiating the progressive gene silencing described in our epigenetic dysfunction model (Gilbert and Liu, 2012). Compounds that simultaneously inhibit carbohydrate digestion and prevent epigenetic silencing could break this pathological cycle at its origin.

Current diabetes management relies on separate targeting of individual pathways, requiring multiple medications with distinct mechanisms and side effect profiles. Single agents capable of dual-target activity could provide superior therapeutic outcomes through pathway synergies while simplifying treatment regimens (Lam et al., 2023). The extensive safety data available for well-characterized metabolic enzyme inhibitors significantly reduces development risks compared to entirely novel synthetic compounds (Paul and Raza, 2024).

The repurposing strategy offers advantages for early intervention during prediabetic stages when epigenetic dysfunction remains reversible. Rather than waiting for overt diabetes to develop and then managing symptoms, dual-target compounds could prevent disease progression by addressing both immediate glucose control and long-term epigenetic preservation. This represents a paradigm shift from reactive symptom management to proactive mechanism-based prevention.

### 5.3 Strategic implementation of cross-target screening

Among our 100 systematically selected metabolic enzyme inhibitors, several categories show promise for cross-target screening. Polyphenolic compounds, which constitute over half of our collection, possess structural features commonly associated with both enzyme inhibition and epigenetic modulation. Alkaloids like berberine have already demonstrated dual activities, suggesting that related structural analogs may exhibit similar cross-target potential (Yin et al., 2008). The identification of additional dual-target candidates requires systematic computational screening followed by experimental validation. Rather than screening all 100 compounds, our approach focuses on those with structural features most likely to achieve cross-target activity, combined with compounds from chemical classes already showing dual activities. This targeted strategy maximizes the probability of discovering therapeutically relevant dual-target candidates while efficiently utilizing research resources.

### 5.4 Proposed mechanistic cascade for insulin resistance reversal

The dual-target inhibition of DNMT1 and HDAC3 by lead compounds initiates a sequential mechanistic cascade to restore metabolic homeostasis:

Direct Enzyme Blockade: High-affinity binding to DNMT1 and HDAC3 active sites directly inhibits their catalytic functions, preventing DNA hypermethylation and promoting histone hyperacetylation.

Chromatin Remodeling: The concerted reduction in CpG methylation and increase in histone acetylation marks (e.g., H3K9ac) at promoters of silenced metabolic genes (e.g., *IRS-1*, GLUT4, INS) transforms the local chromatin from a repressive to a transcriptionally permissive state.

Transcriptional Reactivation: The open chromatin configuration facilitates the binding of RNA polymerase II and essential transcription factors (e.g., PDX-1, MafA), restoring the expression of insulin signaling components.

Phenotypic Recovery: The renewed synthesis of functional proteins re-sensitizes tissues to insulin, culminating in improved systemic glucose uptake, normalized hepatic glucose output, and preserved β-cell function, thereby breaking the vicious cycle of epigenetic-metabolic dysfunction.

## 6 A computational proof-of-concept

To demonstrate the potential of cross-target therapeutic approaches, computational screening was employed to evaluate established α-amylase and α-glucosidase inhibitors against key epigenetic regulators DNMT1 and HDAC3. Molecular docking, a computational technique that predicts how small molecules bind to protein targets, serves as a valuable screening tool for identifying promising candidates before experimental validation (Agu et al., 2023). This computational approach was utilized to illustrate the feasibility of identifying dual-target activities within existing therapeutic libraries, rather than to definitively establish new drug candidates. The analysis demonstrates that modern computational tools can effectively screen metabolic inhibitor libraries for potential epigenetic activities, providing researchers with rational guidance for selecting compounds worthy of experimental investigation.

### 6.1 Computational methodology

Molecular docking analysis was performed using established protocols by Iqbal et al. (Iqbal et al., 2025) to ensure reproducible and comparable results across different target classes. Target protein structures were obtained from the Protein Data Bank https://www.rcsb.org/ accessed on 10 July 2025: HDAC3 (PDB ID: 4A69) and human DNMT1 (PDB ID: 3PTA, crystal structure of human DNMT1(646–1600) in complex with DNA). These structures were selected based on their high resolution and appropriate ligand-bound conformations that represent catalytically relevant states. Following protein preparation, energy minimization was performed using SwissPDBViewer (SPDBV v4.1.0) with the GROMOS96 43B1 force field to relieve steric clashes and optimize hydrogen bonding networks, achieving convergence at 0.01 kcal/mol/Å. This step ensured stable protein conformations before docking analysis. Particular attention was given to maintaining proper zinc coordination geometry in HDAC3 and preserving the S-adenosyl-L-methionine cofactor interactions in DNMT1, as these features are critical for accurate binding predictions.

Binding pockets were defined using the PyRx software’s cavity detection algorithm centered on the known active sites from literature. For HDAC3 (PDB: 4A69), the grid box encompassed the zinc-coordinated catalytic pocket including residues Arg345, Gln71, Gly99, Leu100, Phe101 (Chain B) as confirmed in our docking results. For DNMT1 (PDB: 3PTA), the pocket included the SAM-binding region with residues Arg1363, Ser1034, Glu1025, Thr1031, and surrounding catalytic sites. Grid dimensions were optimized at 40 × 40 × 40 grid points with 0.375 Å spacing.

PyRx software, as described by Dallakyan and Olson (2015) was employed to simulate the binding interactions between the ligands of interest and the active sites of the prepared receptor proteins. Visualization of the interactions between the receptor proteins and key active compounds was performed using Discovery Studio 21.1.0.0 software.

#### 6.1.1 Drug scanning through pharmacokinetics parameters

Evaluating druggability is essential for assessing the potential of a compound as a drug candidate. The druggability of the top compounds was analyzed using SwissADME (Daina et al., 2017). Additionally, the ADMETlab 2.0 online server was employed to evaluate pharmacokinetic and pharmacodynamic properties, including absorption, distribution, metabolism, excretion, and toxicity (ADMET) profiling, as described by Iqbal et al. (2025) Key parameters such as blood–brain barrier permeability, carcinogenicity, skin sensitization, Ames toxicity, and Caco-2 permeability were examined to predict the clinical potential of the compounds.

### 6.2 Molecular docking results: identification of dual-target candidates

The computational screening of 100 natural metabolic enzyme inhibitors against DNMT1 and HDAC3 successfully identified compounds with promising dual-target binding characteristics. The top 10 candidates were prioritized through a sequential filter: first by superior dual-target binding affinity, then by favorable ADMET properties, and finally by structural diversity and clinical relevance (Table 3).

**TABLE 3:** Binding scores of top 10 compounds with histone deacetylase 3 (4A69) and DNA Methyl transferase 1 (3PTA) protein receptor.

Name	PubChem ID	Binding affinity (kcal/mol) with 4A69	Interacting amino acids with 4A69	Binding affinity (kcal/mol) with 3PTA	Interacting amino acids with 3PTA
Kotalanol	42632210	−9.8	ArgA:345, ArgB:72, ValB:73, GlyB:99, LeuB:100, PheB:101, and GluA:347	−10.2	ArgA:1363, SerA:1034, GluA:1025, AsnA:1026, ThrA:1031, ThrA:1364, AsnA:984, and GluA:989
Orientin	5281675	−9.6	LysC:474, LysC:475, ThrC:473, LysB:159, GlnB:506 and IleC:501	−10.1	SerA:1030, SerA:953, ThrA:1031, ThrA:1366, and ArgA:1368
Mangiferin	5281647	−8.5	ArgA:345, GlnB:71, GlyB:99, LeuB:100, PheB:97, PheB:101, and IleA:346	−9.0	ArgA:1363, ThrA:1366, AspA:1369, AspA:1520, ArgA:1368, ThrA:1031, and AlaA:987
Didymin	16760075	−9.4	AsnA:348, GluA:347, GlnB:71, LeuB:70, GlyB:99, ValB:73, LeuB:100, GlyD:452, LeuD:453, ProB:98, ProB:75, and PheB:97	−9.9	ArgA:1437, AlaA:1511, ArgA:1538, SerA:1524, ArgA:1337, GluA:703, ArgA:1311, AspA:701, AsnA:1270, GluA:704, ThrA:1525, and AspA:707
Chicoric acid	5281764	−9.2	LysB:159, LysC:475, ThrC:473, TyrB:160, TyrC:470, ArgC:441, PheC:440, and PheC:444	−9.1	GluA:704, AspA:707, ArgA:1337, ThrA:1309, SerA:1524, ArgA:1538
Eriocitrim	83489	−9.5	AsnD:450, GlyD:452, LeuD:453, ThrB:76, ProB:75, ProB:98, SerB:74, GlyB:99, LeuB:70, and GlnB:71	−9.9	AsnA:1270, AspA:701, GluA:704, ThrA:1525, AsnA:984, ArgA:1337, PheA:1522, ArgA:1311, SerA:1524, and ArgA:1538
Ferulic acid	445858	−8.1	TyrC:470, TyrB:160, GlnB:506, ThrC:473, and PheC:444	−9.7	ArgA:1310, ArgA:1312, GlyA:1577, GluA:698, ThrA:1526, AspA:700, AlaA:699, and ValA:1268
Isoorientin	114776	−9.2	GluA:347, ThrD:462, AlaD:455, LeuD:458, GluD:459, and ProB:75	−8.1	GlyA:1058, ArgA:1356, GluA:1329, GluA:1591, and AsnA:1081
Repandusinic acid A	147900	−9.8	ArgA:345, ArgB:72, AsnA:270, AsnB:77, GlnB:71, PheB:97, PheB:101, LeuB:100, and AspA:228	−9.2	GluA:1518, GlyA:1433, ArgA:1337, GluA:704, AspA:701, GlyA:1512, ArgA:1538, PheA:1522, ArgA:1311, and AspA:707
Corymbiferin	71437983	−8.4	TyrC:470, LysC:474, LysB:159, and GolB:506	−8.9	yrA:863, AlaA:799, GluA:813, ThrA:801, GlyA:806, and SerA:809

Kotalanol emerged as the most promising dual-target candidate, exhibiting remarkable binding affinities of −9.8 kcal/mol with HDAC3 and -10.2 kcal/mol with DNMT1. The compound formed stable interactions with ArgA:345, ArgB:72, ValB:73, GlyB:99, LeuB:100, PheB:101, and GluA:347 in HDAC3, while engaging ArgA:1363, SerA:1034, GluA:1025, AsnA:1026, ThrA:1031, ThrA:1364, AsnA:984, and GluA:989 in DNMT1. Orientin demonstrated strong interactions with both targets (−9.6 kcal/mol with HDAC3 and -10.1 kcal/mol with DNMT1), followed by compounds such as Didymin, Eriocitrin, and Repandusinic acid A, all showing favorable binding characteristics across both epigenetic enzymes.

For benchmarking, the binding affinities of the screened compounds were compared with established positive controls: Trichostatin A for HDAC3 (−7.8 kcal/mol) and 5-Azacytidine for DNMT1 (−8.1 kcal/mol). Notably, natural compounds such as Kotalanol (−9.8 and −10.2 kcal/mol) and Orientin (−9.6 and −10.1 kcal/mol) demonstrated stronger affinities than these references, reinforcing their potential as dual-target inhibitors.

### 6.3 Binding pattern analysis

Figure 7 illustrates the comprehensive binding patterns of Kotalanol with both HDAC3 and DNMT1, demonstrating the compound’s ability to form stable interactions within the active sites of both epigenetic enzymes. The 2D and 3D visualization reveals the specific amino acid contacts and binding orientations that contribute to the favorable binding energies observed.

**FIGURE 7:**
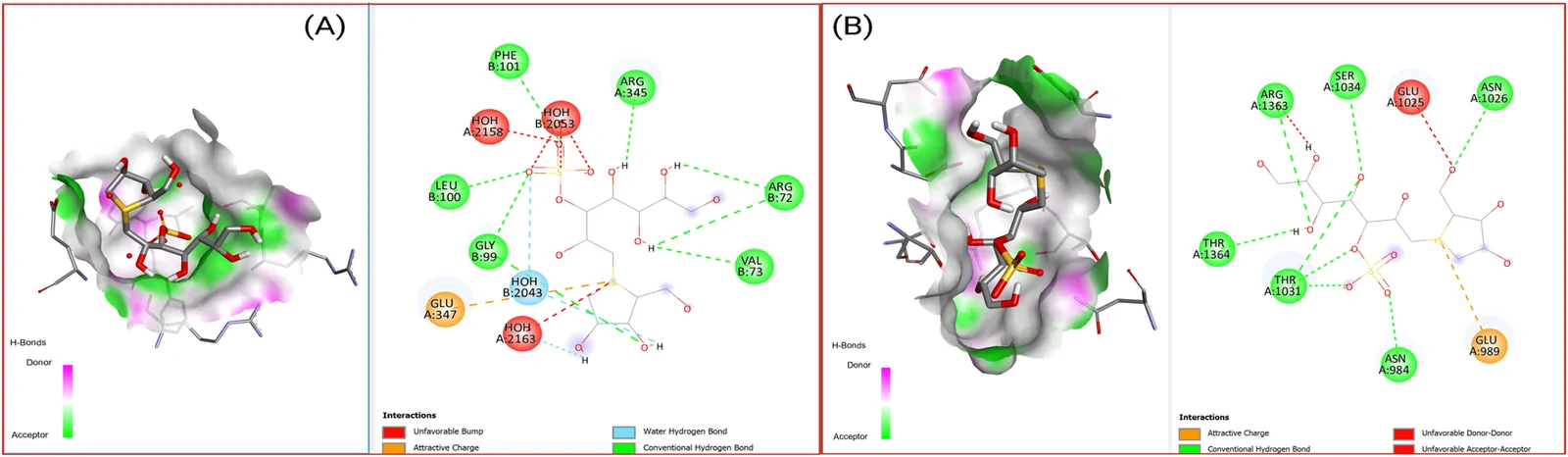
2D Interaction and 3D binding pattern of kotalanol **(A)** with histone deacetylase 3 (4A69) and **(B)** DNA Methyl transferase 1 (3PTA) protein receptor.

Figure 8 presents the binding characteristics of Orientin with both target proteins, showcasing similar dual-target binding capability. The molecular interactions demonstrate how these natural compounds can potentially modulate epigenetic enzyme activity through direct active site engagement, providing mechanistic rationale for their therapeutic potential in insulin resistance treatment.

**FIGURE 8:**
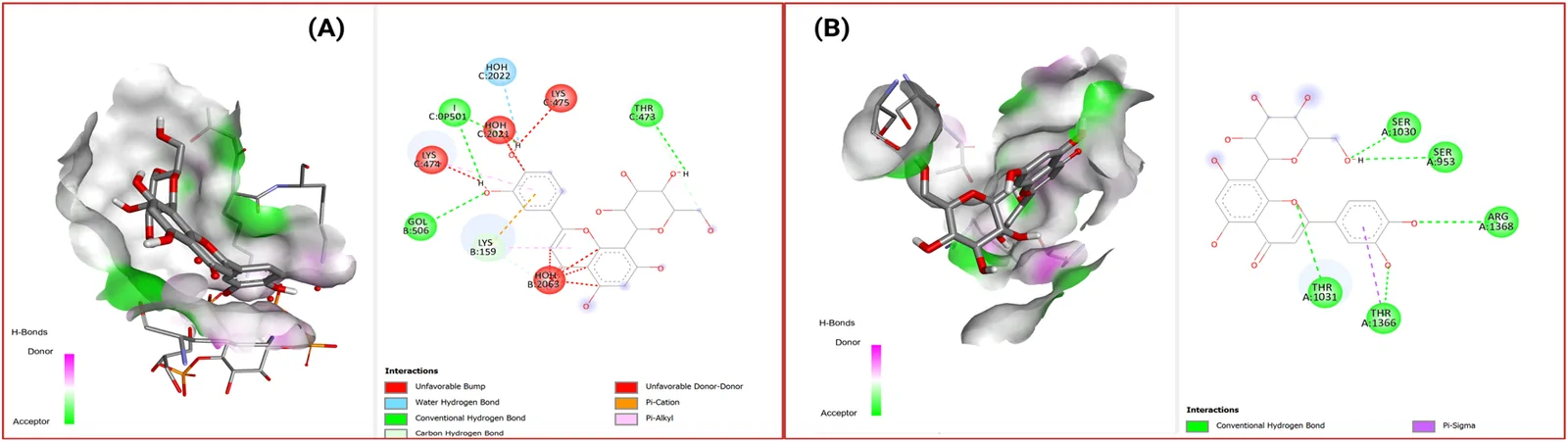
2D Interaction and 3D binding pattern of orientin with **(A)** histone deacetylase 3 (4A69) and **(B)** DNA Methyl transferase 1 (3PTA) protein receptor.

The diversity of binding interactions is further illustrated in Figure 9, which displays the binding patterns of eight selected compounds (Mangiferin, Didymin, Chicoric acid, Eriocitrin, Ferulic acid, Isoorientin, Repandusinic acid A, and Corymbiferin) with DNMT1.

**FIGURE 9:**
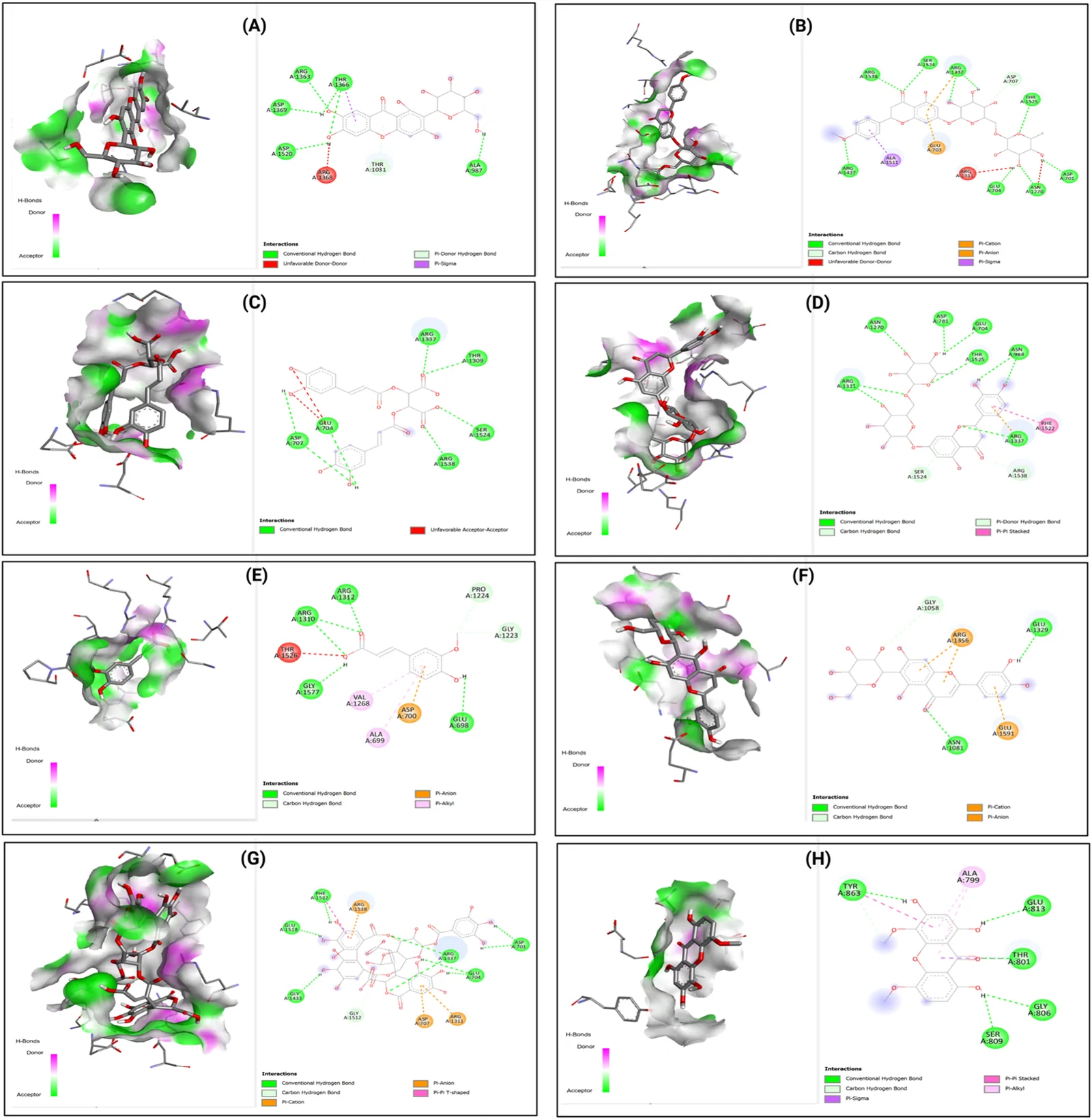
2D Interaction and 3D binding Pattern with DNA Methyl transferase 1 (3PTA) protein receptor as a receptor **(A)** Mangiferin **(B)** Didymin **(C)** Chicoric acid **(D)** Eriocitrim **(E)** Ferulic acid **(F)** Isoorientin **(G)** Repandusinic acid A **(H)** Corymbiferin.

Figure 10 similarly shows the binding patterns of these compounds with HDAC3, demonstrating the versatility of these natural products in accommodating different active site architectures.

**FIGURE 10:**
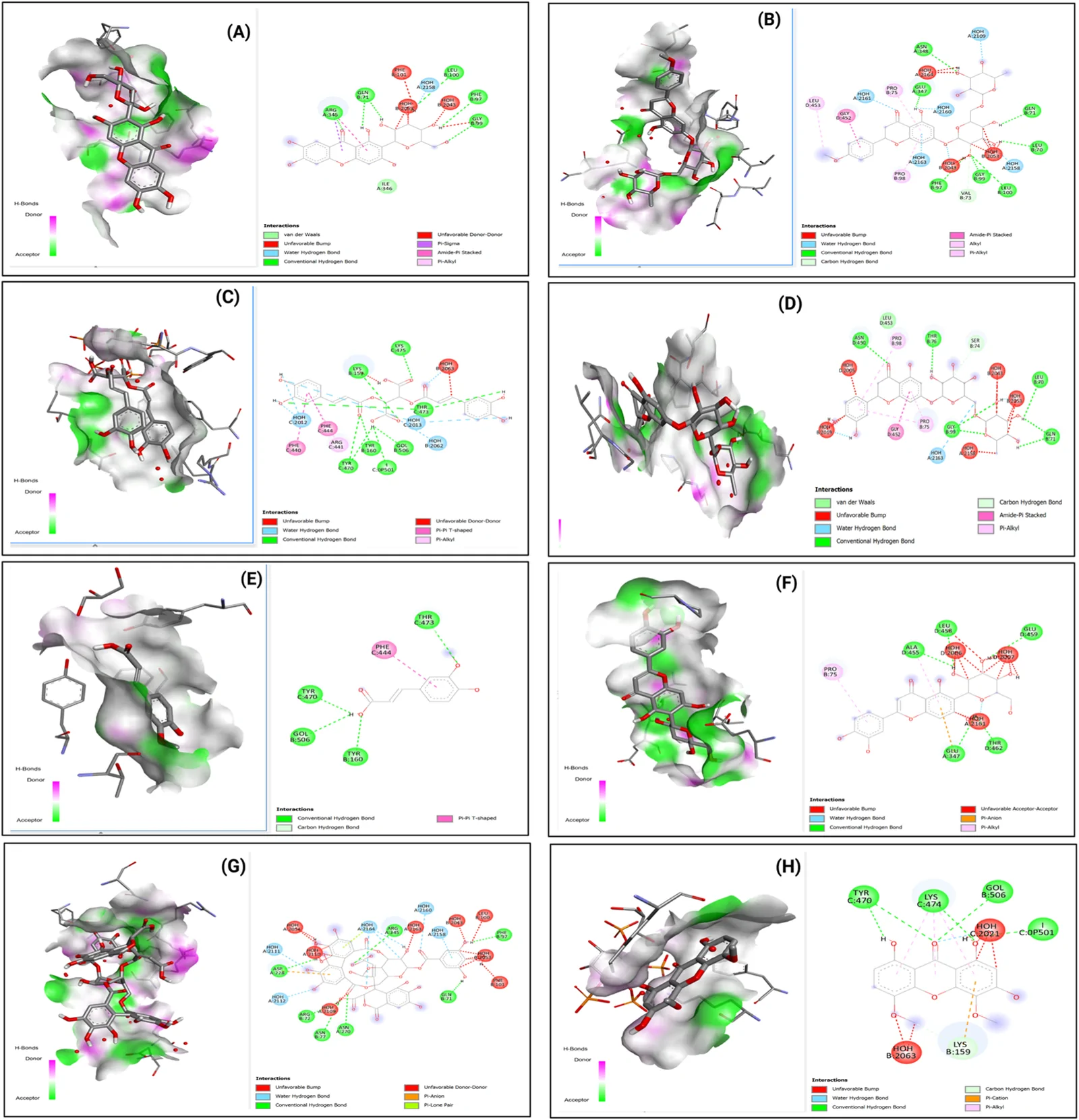
2D Interaction and 3D binding Pattern with histone deacetylase 3 (4A69) protein receptor **(A)** Mangiferin **(B)** Didymin **(C)** Chicoric acid **(D)** Eriocitrim **(E)** Ferulic acid **(F)** Isoorientin **(G)** Repandusinic acid A **(H)** Corymbiferin as a receptor.

### 6.4 ADMET profile assessment

The pharmacological potential of the top-ranked compounds numbered as (1) Kotalanol, (2) Orientin, (3) Mangiferin, (4) Didymin, (5) Chicoric acid, (6) Eriocitrin, (7) Ferulic acid, (8) Isoorientin, (9) Repandusinic acid A, and (10) Corymbiferin was evaluated through comprehensive ADMET profiling using ADMETLab 2.0 (Table 4). The analysis revealed that several compounds exhibited favorable pharmacokinetic characteristics suitable for therapeutic development (Ahmad et al., 2023). Most compounds demonstrated acceptable human intestinal absorption (HIA) properties, with compounds like Kotalanol, Orientin, and Eriocitrin showing good to excellent absorption potential.

**TABLE 4:** ADMET-related drug-like parameters of the selected compounds.

Compounds											
	01	02	03	04	05	06	07	08	09	1010 10	
Absorption and Distribution											
BBB	---	---	---	---	---	---	---	---	---	---	
HIA	+++	++	++	-	++	+++	--	+	+	---	
Caco-2 Permeability	−6.322	−6.208	−6.235	−6.443	−6.955	−6.672	−4.989	−6.239	−8.143	−5.216	
Pgp inhibitor	---	---	---	---	---	---	---	---	---	--	
Pgp substrate	---	--	++	-	---	--	---	--	---	+++	
Volume of Distribution (Vd)	−0.593	−0.011	−0.087	−0.293	−0.302	−0.17	−0.727	−0.06	−0.324	−0.433	
Plasma Protein Binding (PPB)	26.3%	84.4%	81.8%	84.2%	75.0%	80.7%	68.6%	84.6%	59.0%	96.2%	
Excretion											
CLplasma	1.341	3.933	3.951	1.624	5.34	1.442	8.359	4.426	1.122	3.042	
T1/2	2.014	4.944	4.548	4.133	1.936	4.847	1.698	4.489	5.365	1.664	
Metabolism											
CYP1A2 inhibitor	---	-	---	---	--	---	---	--	---	+++	
CYP1A2 substrate	---	---	---	---	---	---	---	---	---	+++	
CYP2C19 inhibitor	---	---	---	---	---	---	---	---	---	---	
CYP2C19 substrate	---	---	---	---	---	---	---	---	---	---	
CYP2C9 inhibitor	--	---	---	---	---	---	---	---	---	---	
CYP2C9 substrate	---	---	---	---	---	---	--	---	---	-	
CYP2D6 inhibitor	--	---	---	---	---	---	---	---	---	---	
CYP2D6 substrate	---	---	---	---	---	---	---	---	---	+++	
CYP3A4 inhibitor	---	--	---	---	---	---	---	-	---	+++	
CYP3A4 substrate	---	---	---	---	---	---	---	---	---	---	
CYP2B6 inhibitor	---	---	---	---	+++	+++	---	---	---	-	
CYP2B6 substrate	---	---	---	---	---	---	---	---	---	---	
CYP2C8 inhibitor	+	+++	+++	--	+++	++	++	+++	+	++	
HLM Stability	---	--	---	--	--	+	--	---	---	++	
Toxicity											
hERG Blockers	0.001	0.011	0.016	0.064	0.02	0.029	0.036	0.014	0.0	0.03	
Carcinogenicity	0.666	0.257	0.283	0.025	0.118	0.025	0.249	0.194	0.027	0.679	
Human Hepatotoxicity	0.559	0.505	0.499	0.684	0.551	0.72	0.702	0.445	0.331	0.399	
AMES Toxicity	0.696	0.838	0.846	0.952	0.308	0.916	0.252	0.803	0.713	0.69	
Drug Likeness rules											
Lipinski Rule	No	No	No	No	No	No	Yes	No	No	Yes	
Pfizer Rule	Yes	Yes	Yes	Yes	Yes	Yes	Yes	Yes	Yes	Yes	

BBB, Blood-Brain Barrier; HIA, human intestinal absorption; CLplasma, Plasma Clearance; T1/2, Half-Life. for classification endpoints, prediction probability values are represented by the following symbols: 0–0.1 (---), 0.1–0.3 (--), 0.3–0.5 (−), 0.5–0.7 (+), 0.7–0.9 (++), and 0.9–1.0 (+++).

Caco-2 permeability values indicated reasonable membrane permeation characteristics across the selected compounds, while plasma protein binding ranged from moderate to high levels, suggesting adequate distribution properties. The compounds showed varied but generally manageable cytochrome P450 enzyme interactions, with most displaying minimal inhibi\

Tion profiles that would reduce potential drug-drug interactions. Toxicity profiling revealed favorable safety characteristics for most compounds, with low carcinogenicity predictions and acceptable hepatotoxicity profiles. The hERG blocker assessment indicated minimal cardiotoxicity risk for most compounds, while Ame’s toxicity predictions suggested limited mutagenic potential.

The comprehensive ADMET evaluation indicates that these natural compounds possess pharmaceutical potential as dual-target therapeutics, with profiles suggesting they could serve as lead compounds for further optimization. The favorable pharmacokinetic parameters, combined with their natural origin and established safety profiles from traditional use, position these compounds as promising candidates for experimental validation and potential clinical development. The integration of binding affinity data with pharmacological profiles provides a robust foundation for prioritizing compounds for subsequent *in vitro* and *in vivo* validation studies, demonstrating the feasibility of cross-target repurposing approaches for insulin resistance therapeutics.

## 7 Future perspectives and therapeutic challenges

The translation of computational dual-target predictions into clinical reality requires systematic experimental validation through integrated cellular models, as outlined in Figure 11, which demonstrates a comprehensive framework utilizing multiple cell lines (HepG2, C2C12, 3T3-L1, and MIN6) to simultaneously assess metabolic enzyme inhibition and epigenetic modulation activities of identified compounds (Zyoud, 2024). While compounds like berberine and curcumin have independent validation for DNMT1 and HDAC3 inhibition respectively, our specific lead compounds kotalanol, orientin, and chicoric acid currently lack experimental confirmation of dual epigenetic activities. Priority validation experiments should include: (1) Direct enzymatic assays determining IC50 values against purified DNMT1 and HDAC3; (2) Methylation-specific PCR and ChIP-qPCR to confirm gene reactivation and histone acetylation changes; (3) Western blot analysis of IRS-1, GLUT4, and insulin receptor expression in insulin-resistant cell models; (4) *In vivo* validation using db/db diabetic mice measuring both metabolic parameters (HbA1c, OGTT) and epigenetic markers (tissue-specific methylation patterns). Critical challenges include developing novel pharmacokinetic strategies to ensure therapeutic concentrations reach both gastrointestinal sites and peripheral metabolic tissues, establishing regulatory pathways for dual-mechanism therapeutics that incorporate both immediate metabolic endpoints and long-term epigenetic biomarkers, and creating personalized medicine approaches that account for individual variations in baseline epigenetic states and metabolic enzyme expression patterns. Acknowledging the pharmacokinetic limitations common to natural products, the therapeutic translation of our lead compounds will necessitate advanced delivery strategies. To overcome challenges of poor bioavailability, rapid metabolism, and non-specific distribution, we propose the development of nanoparticle-based systems (e.g., polymeric or lipid nanocapsules) for encapsulation. These systems can protect the compounds, enhance their intestinal absorption, and provide passive targeting to metabolically active tissues. Furthermore, active targeting approaches using ligands for receptors highly expressed in insulin-responsive tissues (e.g., liver, muscle) and pancreatic β-cells could be employed to achieve tissue-specific action, thereby maximizing therapeutic efficacy while minimizing potential off-target epigenetic effects. The implementation of these delivery technologies is a non-negotiable prerequisite for transforming these potent dual-target inhibitors into viable therapeutics. The validation of our computational framework, which successfully identified ten natural compounds with promising dual-target binding profiles from a library of 100 metabolic enzyme inhibitors, could establish a replicable methodology for mining existing therapeutic databases to uncover hidden cross-target opportunities, potentially bridging the research gap between metabolic and epigenetic medicine while providing a cost-effective alternative to traditional *de novo* drug discovery approaches for complex diseases requiring multi-pathway intervention.

**FIGURE 11:**
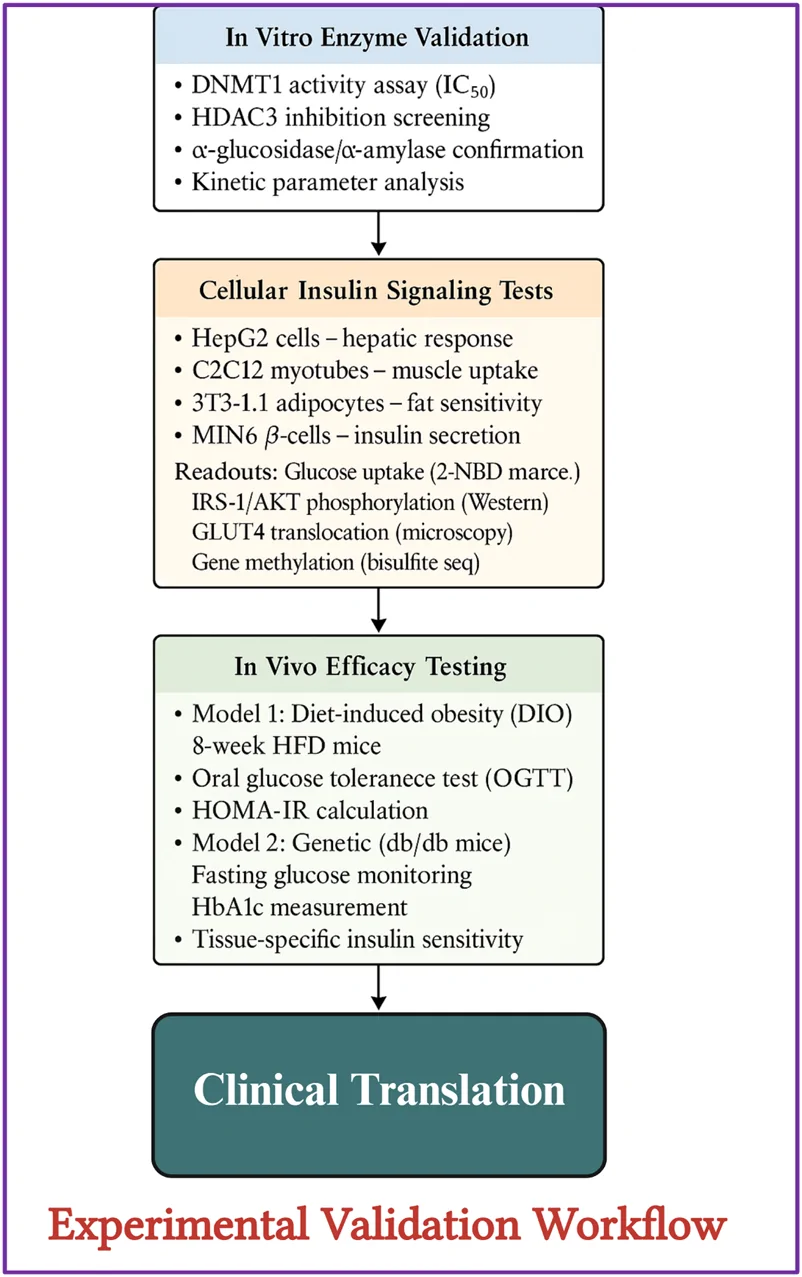
Experimental validation workflow for epigenetic-metabolic dual-target drug discovery. A comprehensive multistep validation strategy for evaluating candidate compounds against epigenetic regulators (DNMT1 and HDAC3) and metabolic enzymes (α-glucosidase, α-amylase). The workflow begins with *in vitro* enzyme assays to determine IC_50_ values and inhibition kinetics, followed by cell-based insulin signaling assays in hepatic (HepG2), muscle (C2C12), adipocyte (3T3-L1), and β-cell (MIN6) models. Cellular readouts include glucose uptake (2-NBDG), IRS-1/AKT pathway activation (Western blot), GLUT4 translocation (microscopy), and gene methylation (bisulfite sequencing). Subsequently, *in vivo* efficacy is tested using diet-induced obese (DIO) and db/db diabetic mouse models to assess glucose tolerance (OGTT), HOMA-IR, HbA1c, and tissue-specific insulin sensitivity. The final phase involves translational potential for clinical applications (Xu et al., 2017).

## 8 Conclusion

This review establishes cross-target repurposing as a transformative approach for insulin resistance therapy. Key findings include: (1) Natural metabolic enzyme inhibitors possess previously unrecognized epigenetic potential, with Kotalanol demonstrating exceptional dual-target binding; (2) Structural conservation between metabolic and epigenetic enzymes enables rational cross-target drug design; (3) Clinically validated compounds like berberine provide proof-of-concept for integrated therapeutic strategies. By simultaneously addressing immediate metabolic dysfunction and underlying epigenetic abnormalities, this approach could shift diabetes treatment from lifelong management to potential reversal. Future efforts should focus on experimental validation of computational predictions and development of optimized dual-target therapeutics for clinical translation.

## Glossary

AGES: Advanced glycation end products
AMP: Adenosine monophosphate
AMPK: AMP-activated protein kinase
ATP: Adenosine triphosphate
G6Pase: Glucose-6-phosphatase
GCK: Glucokinase
GLUT2: Glucose transporter type 2
GLUT4: Glucose transporter type 4
IRS-1: Insulin receptor substrate 1
LDHA: Lactate dehydrogenase A
PEPCK: Phosphoenolpyruvate carboxykinase
PI3K: Phosphatidylinositol 3-kinase
PKM2: Pyruvate kinase M2
PPAR-γ: Peroxisome proliferator-activated receptor gamma
RAGE: Receptor for AGEs
ROS: Reactive oxygen species
SGLT2: Sodium-glucose cotransporter-2
TCA: Tricarboxylic acid cycle
UCP2: Uncoupling protein 2
BET: Bromodomain and Extra-Terminal motif
DNMT1: DNA methyltransferase 1
DNMT3B: DNA methyltransferase 3B
HAT: Histone acetyltransferase
HATs: Histone acetyltransferases
HDAC3: Histone deacetylase 3
HDMs: Histone demethylases
HMTs: Histone methyltransferases
PCAF: P300/CBP-associated factor
PRC: Polycomb repressive complex
SIRT1: Sirtuin 1
UHRF1: Ubiquitin-like, containing PHD and RING finger domains, 1
Beta2: Beta2 transcription factor
FOXO1: Forkhead box O1
MafA: V-maf musculoaponeurotic fibrosarcoma oncogene homolog A
NeuroD1: Neurogenic differentiation 1
NF-κB: Nuclear factor kappa B
Nrf2: Nuclear factor erythroid 2-related factor 2
PDX-1: Pancreatic and duodenal homeobox one
5hmC-DNA: 5-hydroxymethylcytosine DNA
5 mC-DNA: 5-methylcytosine DNA
CpG: Cytosine-phosphate-guanine
H3K27ac: Histone H3 lysine 27 acetylation
H3K9: Histone H3 lysine 9
H3K9ac: Histone H3 lysine 9 acetylation
SAM: S-adenosylmethionine
